# Ni-Induced Novel Multi-Al-Alloys for Efficient and On-Demand Hydrolysis-Based Hydrogen Generation

**DOI:** 10.1021/acsomega.6c01514

**Published:** 2026-05-05

**Authors:** Osman Kahveci, Tuncay Karaaslan, Abdullah Akkaya

**Affiliations:** † Faculty of Science, Physics Department, 52958 Erciyes University, Kayseri 38039, Turkey; ‡ Erciyes University METALION Research Group, Kayseri 38039, Turkey; § Erciyes University Energy Conversion Research and Application Center, Kayseri 38039, Turkey; ∥ Mucur Technical Vocational Schools, Tech. Prog. Department, 187470 Kırşehir Ahi Evran University, Kırşehir 40500, Turkey

## Abstract

Hydrogen generation via aluminum–water reactions offers a safe, on-demand, and sustainable pathway to a clean energy solution for global energy and environmental challenges. However, the formation of passive oxide layers disrupts the reaction kinetics in hydrolysis. In this study, novel quaternary Al–Zn–Si–Ni alloys containing 0.1 and 0.5 wt % Ni were developed to enhance hydrogen generation via hydrolysis in alkaline media. Structural, surface, and electrochemical analyses revealed that Ni addition promotes the formation of Al–Si–Ni and Al–Ni secondary phases, modifies the microstructure morphology, accelerates anodic dissolution, and increases electrochemical activity. The hydrogen generation rate increased 2.3 times for 0.5% Ni content with respect to the Ni-free alloy. The apparent activation energy decreased from 81.91 to 17.28 kJmol^–1^, indicating accelerated reaction kinetics. Electrochemical measurements confirmed lower charge-transfer resistance and decreasing passivation with increasing Ni content and agree with the hydrogen generation results. FESEM image analysis showed that with increasing Ni content, the secondary particle surface phase ratio increased from 0.38% to 1.65%, and the average particle area increased from 5.24 μm^2^ to 13.14 μm^2^. This increased the galvanic activation, thereby increasing the hydrogen generation rate. Overall, Ni doping effectively activates Al alloys by altering their microstructure and electrochemical behavior, enabling a rapid and efficient hydrogen production. These findings provide practical guidance for manufacturing multicomponent aluminum alloys for on-demand hydrolysis-based hydrogen applications.

## Graphical abstract

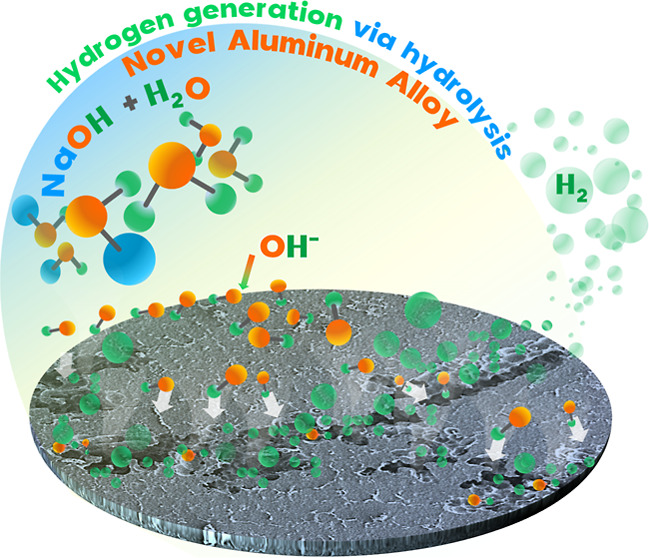

## 1 Introduction

Hydrogen is regarded as a clean and sustainable secondary energy source with abundant resources, high energy density, and high conversion efficiency, which can meet worldwide demands for clean energy.^1–3^ Although steps have been taken from the past to the present to develop hydrogen technologies for various purposes, the common goal has been to replace fossil fuels and mitigate climate change. These aims have motivated research on developing hydrogen technologies. Today, too, the climate agenda appears to be the strongest goal, and there is a growing consensus on the potential of hydrogen technologies. Hydrogen is one of the necessary technological elements to enable the transition to climate-friendly energy sources. Hydrogen technologies are also seen as an opportunity for the development of the industrial sector, which has been significantly disrupted due to reasons such as epidemics and environmental events.^4,5^ The transition process from fossil fuel engines, which are widely used worldwide, to electric motors and from vehicles that pollute the environment to hydrogen-fueled vehicles has accelerated.^6^

Hydrogen is used not only as a fuel but also as an alternative to address the range limitations of electric and hybrid vehicles through fuel cells.^7,8^ However, production costs, transportation, and storage problems constitute significant obstacles to the widespread use of hydrogen’s attractive properties in energy applications.^9^ Although clean hydrogen production costs are higher than those of fossil-based solutions, advances and scientific studies in this area may make it an applicable option in the future. For example, gray hydrogen costs around $0.61–$1.31 per kg, while green hydrogen costs just over $7 per kg.^10^ The production cost of green hydrogen is predicted to decrease, reaching 1.5 $/kg (2050).^4^ A production method that can be used on-site in times of need, thereby minimizing storage and transportation processes, will ensure the effective use of hydrogen technologies. In addition to these features, it would be even more attractive if the hydrogen production rate could be adjusted to meet different power requirements. As a promising production method, hydrogen generation by hydrolysis has come to the fore and has been attracting increasing attention recently.^11,12^ Light metals have good potential as materials for hydrogen generation due to their properties, such as relative safety, environmental friendliness, and the high purity of the produced hydrogen.^13^ Aluminum (Al) has excellent potential to produce hydrogen gas, given a combination of parameters such as high hydrogen capacity, environmental sustainability, abundance of resources, the recyclability of reaction products, and low cost.^14–17^ For example, the price per kg of industrial Al is about $2, about 1/30th the price of another industrial hydrogen-generating material, NaBH_4_.^18^ Water consumption per kilogram of H_2_ produced by the Al-water reaction method (5.96 kg/kgH_2_) comes right after the coal gasification method (2.91 kg/kgH_2_). For example, this value is 21.87 kg/kgH_2_ in steam methane reforming and 305.5 kg/kgH_2_ in biomass gasification.^14^ This feature of the Al-water hydrolysis method is also essential for environmental impact and sustainability. However, oxide film formation in the hydrolysis reaction of aluminum and water limits the reaction rate and hydrogen generation. Influential scientific studies showed that Al activity increased using methods such as ball milling^19–21^ and adding hydrides (e.g., LiH, NaH),^22,23^ salts (e.g., NaCl, MgCl),^24–26^ and metal oxides (e.g., MoO_3_, Bi_2_O_3_, CuO).^21,27^

In addition, many studies exist on alloys in which the activity of aluminum is increased by alloying with low-melting-point metals.^28,29^ The advantages of this method include a repeatable, sustainable production process that is simple and does not require a long time. The desired improvement with the aluminum alloying process is achieved through mechanisms like the formation of intermetallic compounds in the structure^30,31^ and the formation of microgalvanic cells.^32^ Commonly used metals in aluminum alloys for hydrogen production can be listed as Indium (In), Tin (Sn), Bismuth (Bi), Gallium (Ga), Zinc (Zn), Magnesium (Mg), and Copper (Cu).^33–36^ However, not only are these metals used but also different elements are used as a second and third alloying material. Even multicomponent commercial alloys are the subject of research on hydrogen.^37,38^ Nickel (Ni) is one of the metals used as an additive in aluminum alloys. Ni additive studies have generally reported that Ni accelerates the hydrogen generation rate by creating microgalvanic corrosion centers.^39–41^ Metals such as Sn^42^ and Zn,^43^ which have lower melting points than Al, are used in corrosion research of Al. For example, it is known that Zn can enhance the corrosion properties of Al due to its higher mobility and good solubility in an alkaline solution. However, in alkaline solution, Zn causes protective oxide formation on the surface of Al alloys with added Zn. Park et al.^44^ reported that Zn’s passivation effect reduced aluminum’s dissolution rate. It may be possible to break the protective oxide film originating from Zn in alkaline environments using the activation-enhancing effects of Ni. In this way, the effects of Ni on hydrolysis reactions in Zn-doped Al alloys will be explained and may contribute to the understanding gap in this subject. Silicon (Si) has significant effects when used as an additive material in Al alloys. For example, researchers have reported that silicon changes the morphology of Al–Ni alloys from a spherical to a strip-like structure.^45^ The Si addition in Al cast alloys is believed to improve the solubility of additional elements interacting with the dislocation activity of the Al matrix.^46^ Also, Si addition forms intermetallic structures with Al, causes a more uniform elemental distribution, and increases.^47,48^ As mentioned, there are some studies on hydrogen production using Al–Zn–Si alloys. However, these studies have generally focused on Al–Zn or Al–Si binary alloys, and the hydrogen production performances of these alloys have been investigated.^49,50^ Therefore, the catalytic activities of Ni-doped Al–Zn–Si ternary and quaternary alloy structures in hydrogen production reactions have not yet been extensively investigated.

Researchers in this field have generally determined alloys’ hydrogen generation/release rate by investigating closely related corrosion properties. For example, in experiments carried out in salt water or alkaline media, characteristic parameters of materials such as corrosion potential, corrosion current density, and polarization resistance can be determined in this solution. Yang et al.^51^ obtained the highest hydrogen generation rate of 4.95 mL cm^–2^ min^–1^ for the multicomponent alloy using the casting method, and they also performed Tafel polarization analysis of the alloys in NaCl solution and determined the most negative corrosion potential of −1.574 V. In another study, Wang et al.^52^ performed potentiodynamic polarization measurement and electrochemical characterization studies in salt water solution for Zn-containing Al–Si–Cu cast alloys and determined the most negative corrosion potential of −0.835 V. It is sometimes possible to obtain more useful materials suitable for the purpose by combining the different beneficial properties of each component used in alloys. As one of the most relevant studies in this field, Kwon et al.^53^ demonstrated that the Al_3_Ni phase enhances galvanic activation in a binary Al–Ni system and effectively increases the hydrogen production rate compared to pure Al. These findings suggest that Ni addition can create a similar or even richer microstructural effect in Al-based quaternary alloy systems. However, the effect of Ni on phase transformation and hydrogen production kinetics in the presence of additional elements such as Zn and Si, whose beneficial properties have been mentioned, has not yet been systematically investigated. Furthermore, fundamental microstructural and electrochemical findings from such casting-produced alloys have the potential to provide insights into alternative production methods such as mechanical alloying (ball milling) and rapid solidification.

This study aimed to improve the hydrogen generation performance of an Al–Zn alloy in an alkaline environment by leveraging the positive properties of Ni and Si. Thus, these alloys may be useful in hydrogen technologies that require high flow rates and address deficiencies in this field. The hydrogen generation experiments of Al–Zn–Si ternary alloys containing different amounts of Ni in a sodium hydroxide (NaOH) aqueous solution, including the effects of solution temperature, were investigated. In this study, the corrosion properties were investigated by using electrochemical studies to better understand hydrolysis reactions. The surface morphologies of the alloy after corrosion were observed before and after treatment with NaOH. Moreover, the effects of Ni content on the multicomponent Al alloy were analyzed and discussed using X-ray diffraction (XRD), field-emission scanning electron microscopy (FESEM), X-ray energy-dispersive spectroscopy (EDX), and X-ray photoelectron spectroscopy (XPS) characterization techniques.

## 2 Experimental Section

### 2.1 Preparation of Alloys

The hot casting process was first performed on the alloys prepared for hydrogen generation from Al, and the characterization processes were performed on all alloys. Alloy elements Al (99.99% purity), Zn (99.99% purity), Si (99.9% purity), and Ni (99.95% purity) are used to prepare ternary and quaternary alloys. Furnace temperatures are determined by considering the melting points of the alloys. First, aluminum metal was melted in an induction melting furnace (heated to 700 ± 10 °C). After that, an appropriate amount of Zn and Si (the amounts were determined for the corresponding alloy’s rate) was added to the melted Al. This alloy was cooled to room temperature without Ni addition, and the solidification process was carried out, resulting in a cylindrical Al–Zn–Si ternary alloy. For the Ni addition process, the master alloy was heated again in the mold crucibles (700 ± 10 °C), and Ni was added to the melt at the desired rate. To create a homogeneous alloy, the melted alloy is mixed via the effect of induction for 2 min. Melted alloys were cooled to room temperature and solidified, and then, Al–Zn–Si–Ni cylindrical quaternary alloys were obtained. The nominal composition of the alloys was determined as Al–2Zn–1Si–*x*Ni (*x* = 0.0, 0.1, 0.5 wt %).

After that, the cast cylindrical alloys were subjected to metallographic processes for hydrogen generation and characterization. The cylindrical alloys were cut with a precision cutting device (Struers Minitom), and their thicknesses and surface areas were equalized. For equalizing surface roughness, all samples were mechanically polished with 600, 800, 1200, 2400, and 4000 sandpapers. All polished samples used in hydrogen measurements were washed in an ultrasonic bath with ethanol (C_2_H_5_OH) and deionized water (18 MΩ) to remove surface contamination and then dried in N_2_ flow and made ready for the experiment.

### 2.2 Spectroscopic Characterizations

Field-emission scanning electron microscopy (FESEM) (Zeiss Gemini 500) was used to determine the surface morphology and obtain information on the alloy’s chemical composition. X-ray energy-dispersive spectroscopy (EDX) and mapping results were analyzed by using the FESEM-EDX software (EDAX Inc.). Samples were etched with Keller’s reagent^54^ for 10 s for the analysis of phases in FESEM. X-ray diffraction (XRD) patterns were recorded by a Panalytical Inc. X-ray diffraction (XRD) unit (45 keV, 40 mA). The scan range was 20° to 90° (2θ), and the rate was 2°/min. Cu Kα radiation (λ = 1.5406 Å) was an X-ray source. X-ray photoelectron spectroscopy (XPS) measurements were recorded by Specs-FlexMod. An XPS spectrometer was operated at 15 kV and Al Kα radiation (with an energy of 1486.7 eV). The Al 2p, Zn 2s, O 1s, Ni 2p, and Si 2p plots were obtained by taking the average spectra of five scans for each element, and Gaussian–Lorentzian line shapes were used for deconvolution of the spectra.

### 2.3 Evaluation of Hydrogen Generation Performance

Samples cut with a 1.5 mm thickness, 1 cm diameter, and equalized surface areas were tested for hydrogen generation in a 3 M NaOH solution. The hydrolysis performance of aluminum was tested in a jacketed and four-neck glass hydrogen reactor at two different temperatures (330 and 340 K). The H_2_ gas released from the reactor passes through a washing bath and a dryer and reaches the two-stage mass flow meter (Line Tech). The instantaneous H_2_ flow rate and total accumulated gas volume were recorded on the computer every second. The reactor was maintained at a set temperature with the help of a liquid circulator (Polyscience Chiller). The temperature value of the reactor was instantaneously recorded to the computer, too, via a data logger (Pico TC-08) using a K-type thermocouple (Figure 1a).

**Figure 1:**
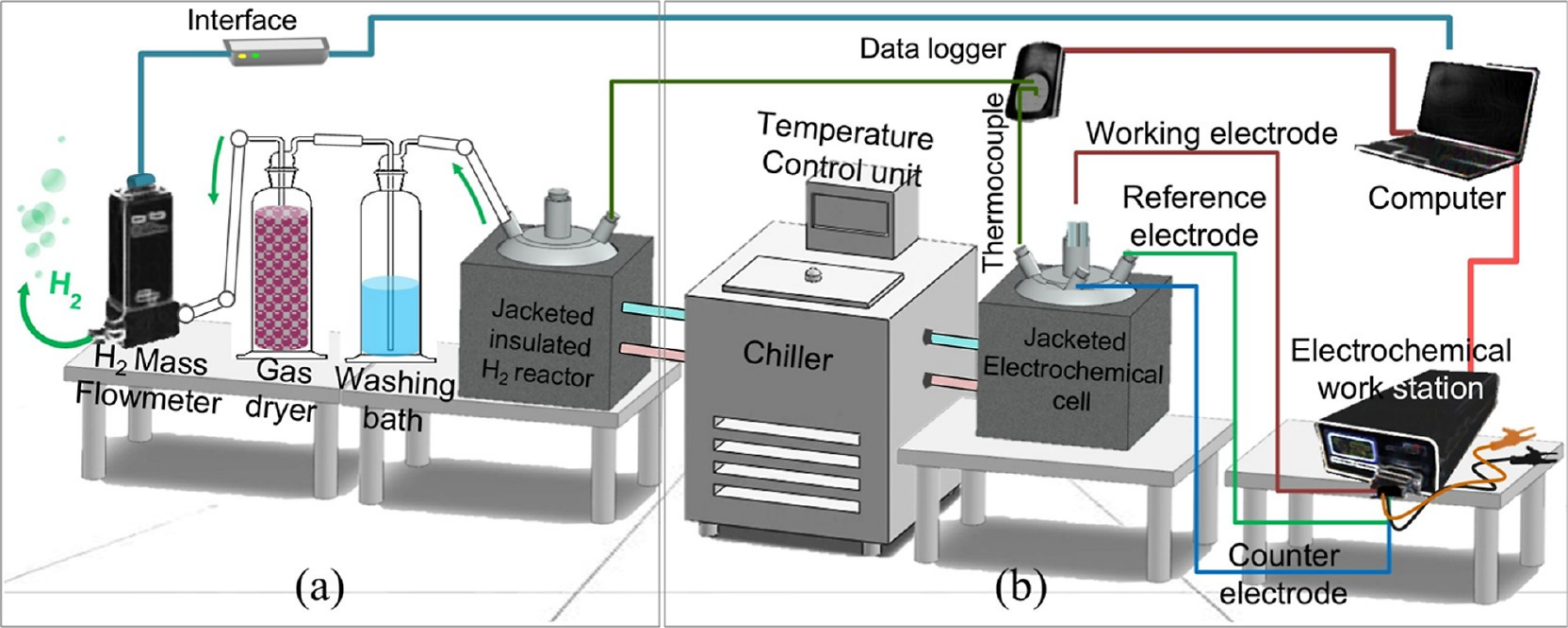
Schematic view of the experimental setup for (a) hydrogen measurement and (b) electrochemical measurement (illustrations and photographs were created by the authors).

### 2.4 Electrochemical Measurements

Alloys were tested for electrochemical measurements in the same environment (3 M NaOH) as that in the hydrogen generation measurements. The measurements were conducted on a Zive SP 1 electrochemical workstation (Wonatech Inc.) (Figure 1b). The reference, counter, and working electrodes used in the three-electrode measurement system were a Ag/AgCl (3 M NaCl) electrode, a Pt sheet (1 cm^2^) electrode, and designed Al alloy electrodes (1 cm^2^ active surface area), respectively. Each experiment was carried out using 50 mL of fresh NaOH solution. Potentiodynamic polarization measurements (Tafel) were carried out in the potential range from −2.2 V to −1 V with a scan rate of 1 mV s^–1^. Electrochemical impedance spectroscopy (EIS) analysis was set to be in the ac signal frequency range of 200 kHz–200 mHz, with a resolution of 1 mV s^–1^, and the ac signal amplitude was 5 mV. The experimental data were modeled using the integrated software ZMAN 2.5, and equivalent circuit analysis was performed. Equivalent circuit modeling was performed by considering the agreement between the experimental and model results for both the phase shift and impedance as a function of frequency. Nyquist and Bode plots are given in the “Electrochemical Analysis” section. Each measurement was performed in a fresh solution after waiting for 180 s to reach equilibrium in the solution. 3 M NaOH was used to ensure that the hydrogen reactor and the corrosion test environment were the same solution. Therefore, the equilibrium time was selected as 180 s due to the reaction of the alloy used with NaOH.

## 3 Results

### 3.1 XRD Analysis

X-ray diffraction patterns of the Al–2%Zn–1%Si (wt %) ternary alloys with different Ni contents are shown in Figure 2. Al–2%Zn–1%Si ternary and 0.5% Ni-doped Al–2%Zn–1%Si quaternary alloy were mainly composed of an Al-rich phase. The dominant α-Al peaks (111), (220), and (311) are common in all alloys (PDF card 00-004-0787). It was observed that the doping atoms (Zn, Si, and Ni) were well dissolved in the melted aluminum, and some Al phases were formed at the end of the alloying procedure. However, due to their low content, no Ni-related peaks were observed in the XRD pattern. This can be explained by the fact that the amount of added Ni (0.1–0.5%) remained below the instrument’s detection limit. Similarly, it has been reported in the literature that low-proportion alloy elements cannot be detected by XRD due to their sparse distribution in the matrix. This may also indicate that Ni elements are present either as dissolved substitutional solids or in negligible amounts.^55^

**Figure 2:**
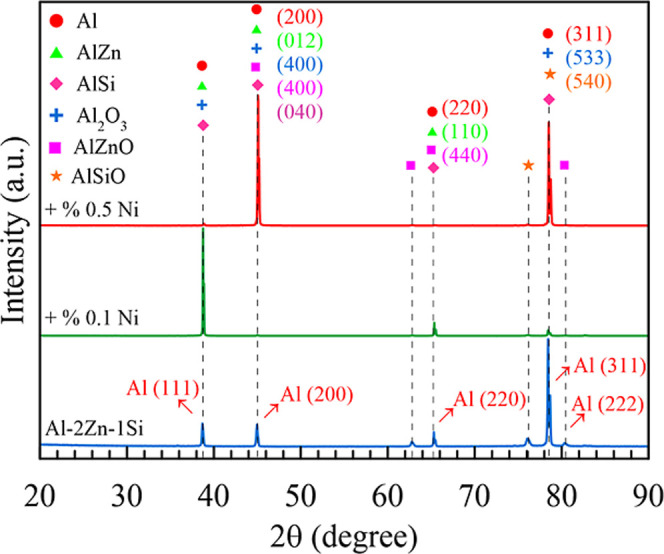
XRD analysis of Al–2%Zn–1%Si ternary alloys with different Ni contents.

In addition, the diffraction peaks revealed slight oxidation, with signals corresponding to Al_2_O_3_ (PDF card 01-079-1558), AlZnO (PDF card 00-001-1146), and AlSiO (PDF card 00-003-0165).

### 3.2 FESEM Analysis before the Hydrolysis Reaction

Figure 3 presents FESEM images of the alloys, magnified phase images, EDX analyses of the phases, and EDX-mapping results before hydrogen generation. The FESEM image of the Al–2%Zn–1%Si (wt %) ternary base alloy exhibited a homogeneous microstructure with no distinct secondary phase particles observed. In the overall image, no distinct bright secondary phase regions were detected within the matrix and the general structure showed a fine network pattern.

**Figure 3:**
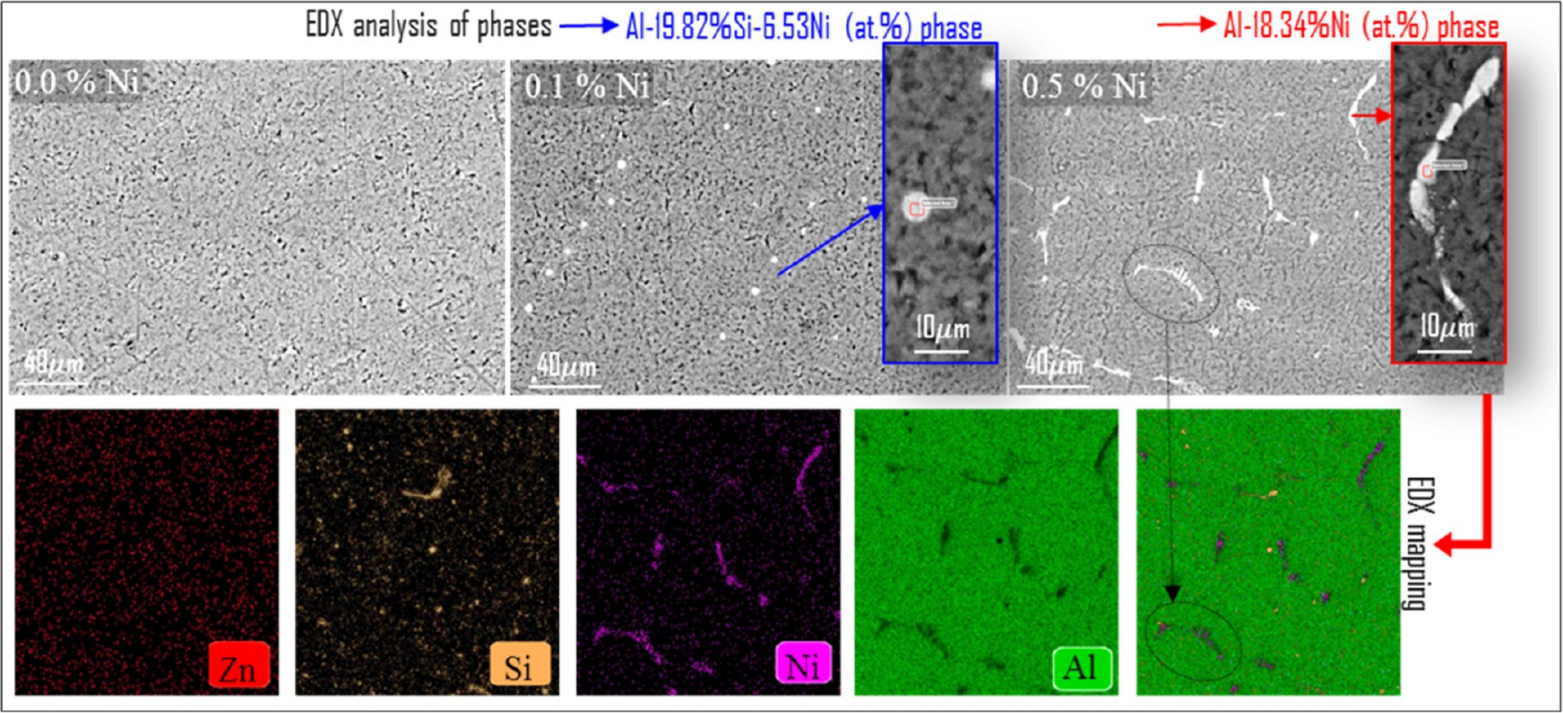
FESEM images of Al–2%Zn–1%Si ternary alloys with different Ni contents before the hydrogen generation and the EDX-mapping result of the 0.5% Ni alloy.

With the addition of 0.1% Ni, homogeneously distributed spherical morphological Al–Si–Ni secondary phase particles were formed within the matrix. Image analysis revealed that these particles had an average length of 3.29 μm, an average area of 5.24 μm^2^, and a surface phase ratio of 0.38% (using ImageJ software). EDX analysis confirmed that these phases had a composition of Al-19.82 atom % Si-6.53 at. % Ni.

With the increase in the Ni content to 0.5%, a significant change in phase composition and morphology was detected. In this case, Si was largely excluded from the phase, and a Ni-enriched Al–Ni binary phase (Al-18.34 at. % Ni) was formed. These phases exhibited a more elongated, finer morphology with an average length of 13.51 μm and an average width of ∼2 μm. From the image analysis, it was determined that the phase ratio increased to 1.65% and the average area to 13.14 μm^2^ (statistical data and explanations regarding the measurement of the phases are provided in detail in Figures S3–S5 and Table S2). The size and shape of the phases are important; the distribution and size of the phases in the structure affect hydrolysis reactions.^56^

The mapping of the 0.5% Ni alloy, which has the highest Ni doping, was performed from the selected area. These images reveal regions rich in both Ni and Si, as well as areas where they are codense. These regions coincided with areas where Ni-enriched second-phase particles were densely observed in the FESEM images. Si mapping showed that it was largely separated from the Ni-rich regions and concentrated in different locations. This finding confirmed that with increasing Ni incorporation, Si was excluded from the second phase and precipitated in separate regions. This phase transformation, triggered by Ni addition, along with the accompanying coarse-grained growth and morphological evolution, is expected to have a decisive effect on the hydrogen production reaction kinetics of the alloys. Zn, on the other hand, appears to be homogeneously dispersed in the Al matrix. It is known that Zn has a wide solid solubility range in the Al–Zn binary system and does not form a separate second phase.^57^ In this respect, the mapping results were obtained in a meaningful framework.

### 3.3 Hydrogen Generation Performance of Alloys

Cylindrical disc-shaped samples, prepared by metallographic processes to have equal surface areas, were subjected to hydrogen generation measurement. The graphs obtained from the experiments performed at two different reactor temperatures (330 and 340 K) are given in Figure 4. At a 330 K reactor temperature, the hydrogen flow rate increases as the Ni doping ratio increases. Similarly, at a 340 K reactor temperature, the highest instantaneous hydrogen flow rate is obtained from the highest 0.5% Ni alloy. Figure 4a for 330 K and Figure 4b for 340 K clearly show these results. Hydrogen flow rates increase until they reach a maximum in nickel-containing alloys but then begin to decrease as the sample gets smaller. In the Al–2%Zn–1%Si alloy, the effects of oxide formation and dissolution on the surface can be clearly seen from the hydrogen flow rate. In this alloy, the hydrogen flow rate is much lower and fluctuates; it takes a long time for the sample to shrink. Figure S1 shows the temperature variation of the reactor over time during hydrogen generation. The effect of the exothermic reaction (Al reacting with a NaOH solution) can be seen from these graphs. The reactor temperature, which starts with 330 K, reaches a maximum value of approximately 331.4 K in 15 min in the 0.0% Ni-doped alloy, while it reaches 333.8 K in 10.8 min in the 0.5% Ni-doped alloy. Similarly, the reactor temperature starting at 340 K reaches 341.6 K in the 0.0% Ni alloy and 343.8 K in the 0.5% Ni alloy in a shorter time. The effect of the Ni content in the Al–2%Zn–1%Si alloy on the increase in the hydrogen flow rate can also be observed at the reactor temperature. Researchers may need to consider the reactor temperature parameter for flow rate control.

**Figure 4:**
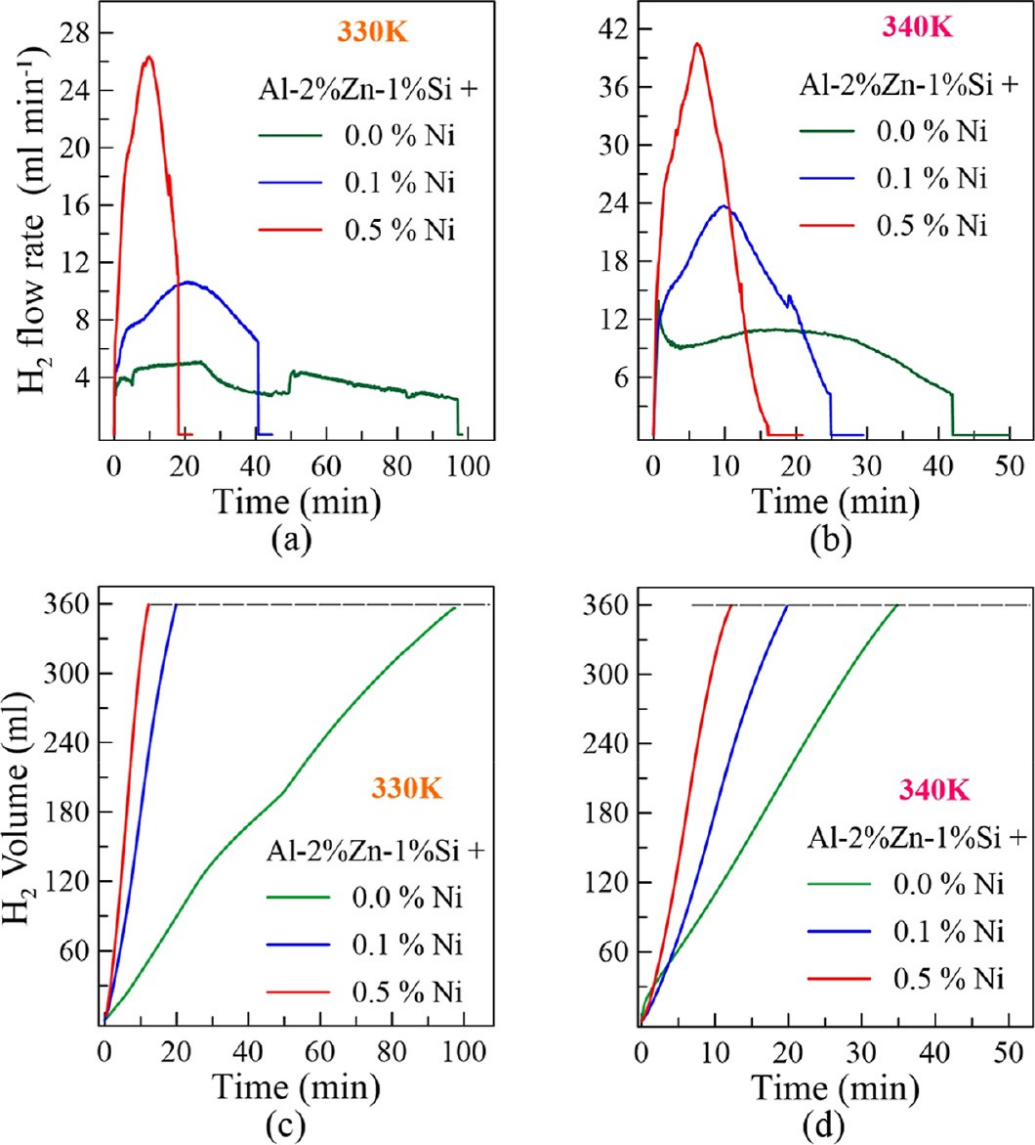
Hydrogen flow rate of alloys, at (a) 330 K and (b) 340 K set temperatures, and time-dependent total hydrogen generation of the alloys for (c) 330 K and (d) 340 K set temperatures.

Figure 4c,d shows the total volume of hydrogen generated at reactor temperatures of 330 and 340 K, respectively. The alloy with the highest Ni content, 0.5% Ni, reaches 360 mL of hydrogen generation at both temperatures. While the 0% Ni alloy reaches 360 mL generation at 330 K in about 98 min, the 0.5% Ni alloy only takes about 18 min. At 340 K, 0.0% Ni reaches 35 min, and 0.5% Ni reaches 12 min. Adding 0.5% Ni makes it possible to reach the targeted amount of hydrogen faster, even at lower temperatures. At 330 K, the 0.5% Ni alloy produces 360 mL of hydrogen in 18 min, and the 0.0% Ni alloy can reach this amount in 35 min, even at 340 K. With the addition of Ni, hydrogen generation becomes possible at lower temperatures and faster rates. The results show that Ni addition in hydrogen generation can save time and energy.

The hydrogen generation rate of the alloys was calculated in two different ways: per mass and per active surface area. The hydrogen generation rate per mass (*V*_g_) was calculated from eq 1.

1 $$V_{g}=\frac{V_{H_{2}}}{\Delta m\times \Delta t}$$

Here, *V*_H_2__ is the total amount of hydrogen produced (mL), Δ*t* is the time taken for production (min), and Δ*m* is the mass consumed (g). The hydrogen generation rate per active surface area (*V*_s_) was also calculated from eq 2.

2 $$V_{s}=\frac{V_{H_{2}}}{S\times \Delta t}$$

where *S* is the initial active surface area (1 cm^2^). Figure 5a,b shows the change in hydrogen generation rate per mass and per active surface area depending on the Ni addition, respectively. Calculations of two different hydrogen generation rates enable an easy comparison with literature values. For example, Li et al.^58^ calculated only the hydrogen evolution rate per active surface area for the Al-7075 alloy, and this value was 0.475 mL cm^–2^ min^–1^. He et al.^59^ calculated the hydrogen generation rate per mass and obtained it as 104 mL cm^–2^ min^–1^ at 50 °C for a multicomponent Al alloy with 8 wt % Cu addition. Liang et al.^60^ systematically investigated the effects of Co, Fe, and Ni additions on the Al–H_2_O reaction. They obtained hydrogen yields of ∼1075 mL g^–1^ with Ni and ∼970 mL g^–1^ with Co at 35 °C; the maximum reaction rates of 5 and 4 mL g^–1^ min^–1^, respectively. For mass-based comparisons, the Ni–Li–B catalytic system yields an average production rate of 0.50 mL min^–1^, outperforming the 0.36 mL min^–1^ rate reported for standard Fe–B catalysts.^61^ Jia et al.^41^ reported a hydrogen generation capacity of 1170 mL g^–1^ at 45 °C for a ball-milled Al/Ni/NaCl mixture (for an optimum Ni: Al ratio of 2:10). Ho and Huang^62^ demonstrated that waste aluminum cans pretreated by planetary ball milling, combined with Ni additives and 0.25 M NaOH, achieved a hydrogen generation rate of 150 mL s^–1^ g^–1^ at 70 °C. Additionally, experimental parameters related to hydrogen generation are given in Table 1.

**Figure 5:**
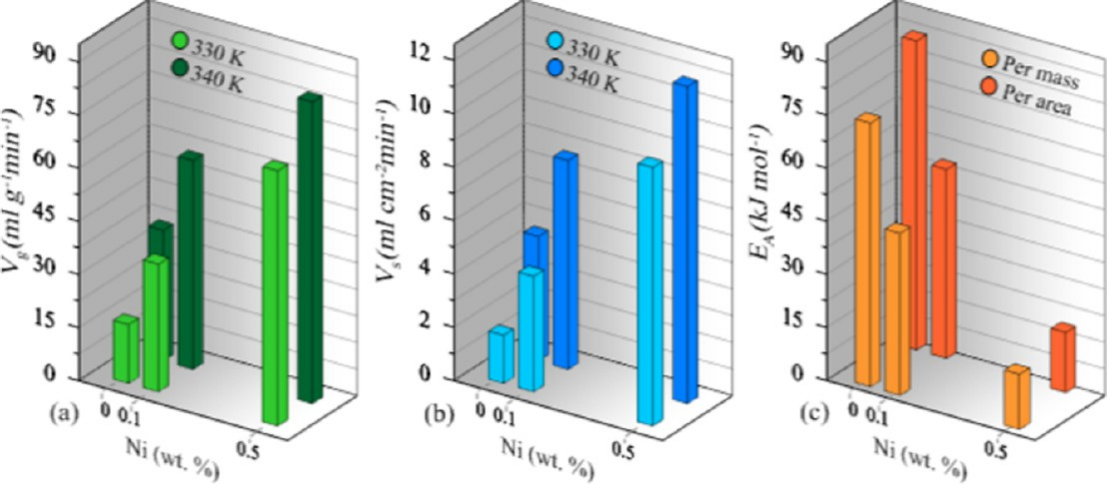
Depending on the Ni addition in the alloy, (a) per mass, (b) per active surface area, hydrogen generation rate, and (c) apparent activation energies.

**Table 1:** Experimental Parameters of Alloys Obtained from Hydrogen Generation Measurements

	initial mass (g)		max. H_2_ flow rate (mL min^–1^)		*V*_g_ (mL g^–1^ min^–1^)		*V*_s_ (mL cm^–2^ min^–1^)		
sample	330 K	340 K	330 K	340 K	330 K	340 K	330 K	340 K	$\overset{®}{E_{A}\;}$ (kJ mol^–1^)
0.0% Ni	0.299	0.298	5.11	11.17	16.59	36.89	1.79	4.66	81.91
0.1% Ni	0.291	0.299	10.66	23.73	36.07	58.94	4.32	7.82	50.59
0.5% Ni	0.293	0.291	26.38	40.49	72.04	85.16	9.68	11.86	17.28

At 340 K, the *V*_s_ value is 4.66 mL cm^–2^ min^–1^ for 0.0% Ni, and it is almost possible to reach this rate at 330 K with a 0.1% Ni contribution. Adding 0.5% Ni makes reaching approximately twice the rate possible, even at lower temperatures. It is possible to obtain similar values in *V*_g_. These data also support that energy and time savings are possible.

### 3.4 Kinetics of the Reaction

The effect of the temperature on the corrosion of aluminum in an alkaline medium was investigated. The alloys’ apparent activation energies (*E*_A_) were calculated to evaluate the effects of Ni addition and temperature. The apparent activation energies for two different corrosion rates, *V*_g_ and *V*_s_, calculated from eqs 1 and 2 in hydrogen generation, were calculated separately using the Arrhenius equation.^63^ Eq 3 for mass *E*_A1_ and eq 4 for areal *E*_A2_ were used.

3 $$\log\left(\frac{V_{g2}}{V_{g1}}\right)=\frac{E_{A1}}{2.303R}\left(\frac{1}{T_{1}}-\frac{1}{T_{2}}\right)$$

4 $$\log\left(\frac{V_{s2}}{V_{s1}}\right)=\frac{E_{A2}}{2.303R}\left(\frac{1}{T_{1}}-\frac{1}{T_{2}}\right)$$

Here, *R* is the universal gas constant, and *T*_1_ = 330 K and *T*_2_ = 340 K are the reaction set temperature values. The change in the calculated values as a result of the Ni addition is shown in Figure 5c. The average values of the apparent activation energies calculated from these two corrosion values are presented in Table 1. $\overset{®}{E_{A}\;}$ Ni doping provides a significant decrease in *E*_A_. The $\overset{®}{E_{A}\;}$ value, which was 81.91 kJ mol^–1^ for the 0.0% Ni alloy, decreased by about one-fifth to 17.28 kJ mol^–1^ for the 0.5% Ni alloy. This result is also highly consistent with the hydrogen flow rates. The reaction kinetic study confirms that Ni accelerates the corrosion of Al under alkaline conditions and enhances the hydrogen generation performance. In addition, the increase in convection currents accelerates the reaction for all samples with increasing temperature.^29^ In the phase analysis, the surface phase ratio of the second-phase particles increased from 0.38% to 1.65%, and the mean area increased from 5.24 to 13.14 μm^2^, while the morphology evolved from a spherical to a more elongated morphological structure. These results indicate that the Ni concentration significantly alters not only the phase composition but also the microstructural architecture, suggesting that the decrease in apparent activation energy (from 81 to 17 kJ mol^–1^) may also be related to the increase in phase size and morphological transformation.

### 3.5 Electrochemical Analyses

Additional analyses were applied to elucidate the reaction mechanisms of the alloys during hydrogen generation. Potentiodynamic polarization measurements of the alloys were performed in 3 M NaOH at room temperature. Tafel polarization curves of the samples are given in Figure 6a. When Al is exposed to an alkaline environment, a series of reactions occur. Kahveci^64^ reported detailed information about these reactions in his experimental work. During the reaction in an alkaline environment, Al dissolves and hydrogen is produced through water reduction. In fact, the reaction is considered undesirable and parasitic for Al-air battery application, and it is necessary to increase the hydrogen generation from Al. The corrosion potential shifts toward positive values as the Ni contribution increases. Around −1.9 V without Ni contribution, the value shifts to approximately −1.4 V with 0.5% Ni contribution. It can be said that the complex structure containing Ni and the intermetallic structure containing Ni can act as a corrosion catalyst. Zhou et al.^65^ have expressed similar results for the Al_2_Cu phase and reported that the potential of the Al alloy containing Cu is more positive than that of pure Al. The more positive corrosion potential may be due to the galvanic corrosion of the connected AlNi/Al system. Thus, pure Al is thought to easily dissolve and act as an anode, while the intermetallic AlNi phase acts as a cathode by shielding. In the images after corrosion, it is clearly seen in Figure 8 that the dissolution in the sample increases. Similar effects of Al-containing intermetallic compounds on the potential and dissolution process of Al have been reported before.^66^ Figure 6b shows the change in polarization resistance (*R*_P_) and corrosion rate (*C*_R_) values with Ni content by weight. The *R*_p_ value of the ternary alloy is 10.88 Ω; this value decreases to 2.80 Ω with the addition of 0.5% Ni. Coordinately, the *C*_R_ value increases more than 4 times from 214.6 mm yr^–1^ to 911.7 mm yr^–1^ with the contribution of 0.5% Ni.

**Figure 6:**
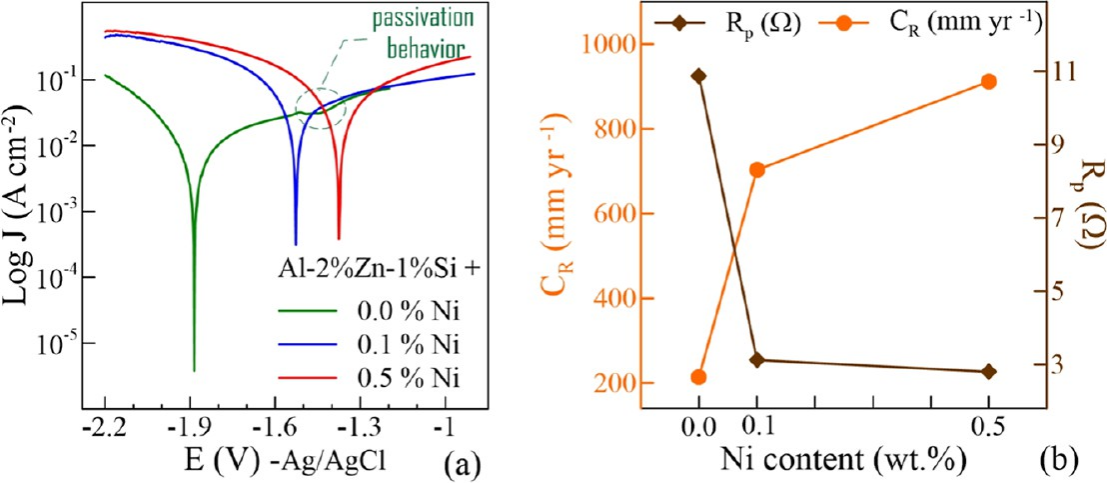
(a) Potentiodynamic polarization curves of ternary and quaternary alloys in 3 M NaOH. (b) Change in polarization resistance (*R*_P_) and corrosion rate (*C*_R_) values with Ni content.

Potentiodynamic polarization results were calculated using IVMAN Main Software (WonATech Co., Ltd.) and are given in Table 2. The formula used to calculate the R_p_ value from the data obtained using Tafel extrapolation is given in eq 5.^64^

5 $$R_{p}=\frac{\beta_{a}\times \beta_{c}}{\left(\beta_{a}+\beta_{c}\right)\times 2.3\times J_{cor}}$$

β_a_ and β_c_ given here are the slopes of the linear lines fitted to the anodic and cathodic sides of the Tafel curve, respectively, and *J*_cor_ is the corrosion current density. The *C*_R_ value was calculated using eq 6.^67^

6 $$C_{R}=K_{R}\frac{J_{cor}}{\rho }E_{w}$$

*K*_R_ is a constant, ρ is the alloy density, and *E*_w_ is the equivalent weight.

**Table 2:** Potentiodynamic Polarization Behavior of the Alloys in a 3 M NaOH Solution

sample	density (g cm^–3^)	*E*_cor_ (V)	*J*_cor_ (mA cm^–2^)	β_a_ (mV)	β_c_ (mV)
0.0% Ni	2.733	–1.885	10.867	754	285
0.1% Ni	2.734	–1.526	27.073	563	211
0.5% Ni	2.753	–1.377	35.070	414	308

Examining the *J*_cor_ values of the samples shows that Ni addition significantly increases the current density. An increase in the *J*_cor_ value indicates a decrease in the corrosion resistance. In Figure 6a, a decrease in the *J*_cor_ occurs at −1.5 V in the Tafel curve of the 0% Ni alloy and stabilizes again at −1.35 V. This behavior in the Tafel curve occurs during the oxidation and dissolution process of the alloy surface.^68^ Passivation behavior is observed in the AlZnSi ternary alloy without Ni. No passivation is observed in other alloys containing Ni in the Tafel curve. Additionally, it is seen that the β_a_ value decreases with the addition of Ni. The small β_a_ value may explain the increased dissolution rate of Al,^69^ resulting in faster hydrogen generation. With the addition of Ni, anodic dissolution accelerates and increases the electrochemical activity of the alloys. Potentiodynamic polarization measurement results appear to be in good agreement with the hydrogen generation results of the samples.

Electrochemical impedance spectroscopy (EIS) was also performed on the alloys. The curves obtained from the equivalent circuit model agree with the experimental data. The Nyquist curve of the alloys is shown in Figure 7a. The frequency-dependent impedance changes and phase on a logarithmic scale are shown in Figure 7b,c, respectively, and the equivalent circuit model is given in Figure 7d. In these figures, some frequency values are marked for the sake of clarity.

**Figure 7:**
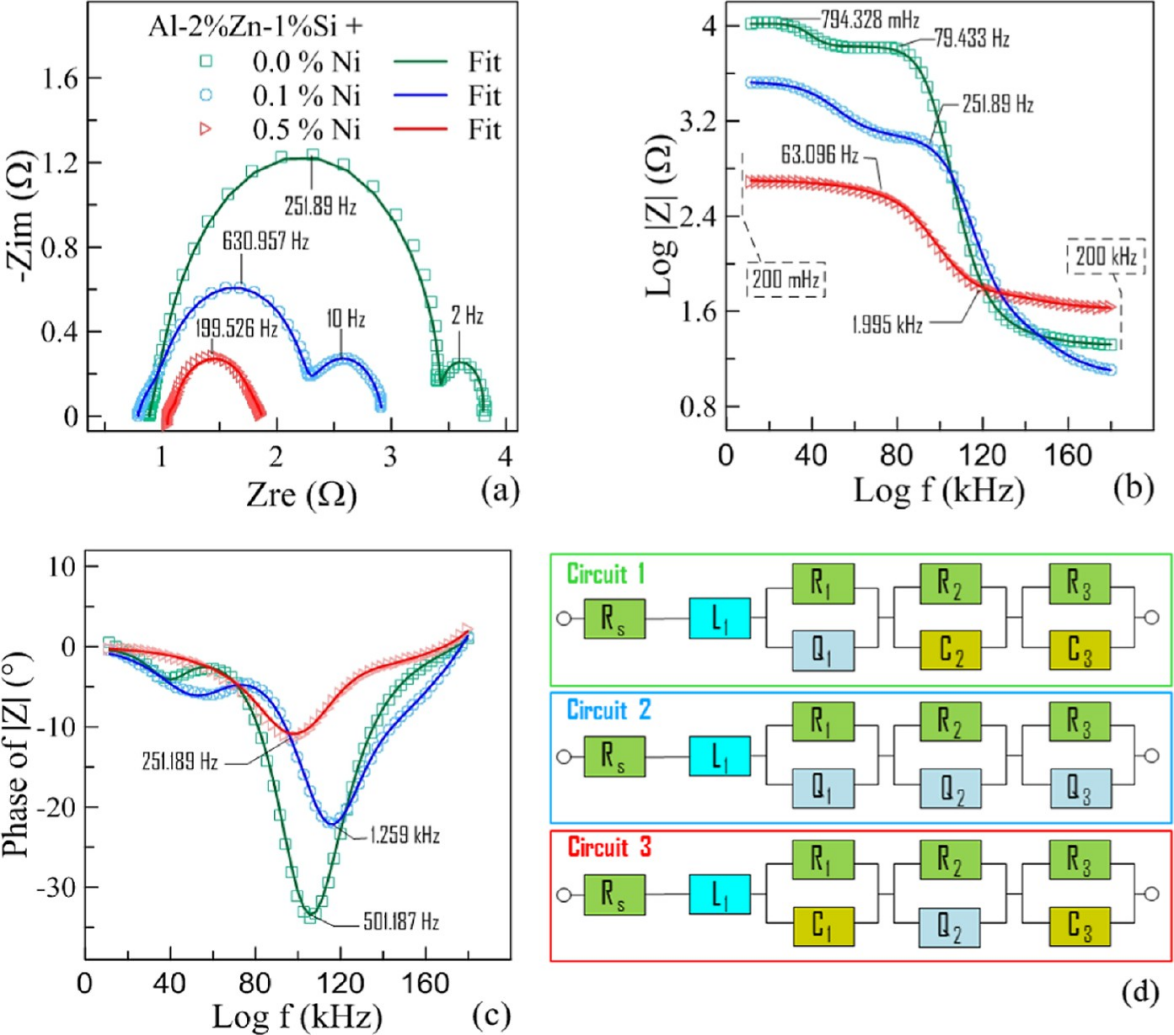
(a) Nyquist plot for different Ni contents of Al–Zn–Si alloys at a 25 °C temperature, (b) Bode plot for |*Z*|, (c) and Bode plot for the phase, and (d) equivalent circuits used for each alloy to fit EIS data consisting of different circuit elements.

The Nyquist curve for the Al–2%Zn–1%Si ternary alloy starts with a slightly pronounced loop in the high-frequency region, takes the form of a semicircular loop in the medium–high frequency region, and has a semicircular loop with a smaller radius in the low-frequency region. The quaternary alloy with 0.1% Ni addition is similar, but the loops have some differences. In the quaternary alloy with 0.5% Ni addition, the loop at high frequency is slightly more pronounced, while the capacitive loop at low frequency is less pronounced. The loop at high frequency is considered to be the inductive loop related to hydrogen evolution. L_1_ is defined as the parameter responsible for hydrogen evolution.^28,70^ The inductive behavior is observed when the oxide layer breaks down locally; this typically occurs due to the presence of current during anodic dissolution, leading to hydrogen evolution.^71^ In the hydrogen evolution reaction (HER), surface bubble formation and reduction may create a delayed (inductive) effect on the system response. The presence of an anodic current also supports the presence of galvanic couples within the microstructure. FESEM mapping results support this behavior (Figure 10). An additional capacitive effect of the bubbles formed during hydrogen generation is represented by *Q*_1_ or *C*_1_, and *R*_1_ is the associated resistance. Thus, *R*_1_ represents ohmic barriers, reflecting a temporary surface resistance created by bubbles^72^ due to gas dynamics released during the HER. Q is the constant-phase element and shows a deviation from the ideal capacitor. The model parameter represented by “*n*” can be defined as an indicator of the approximation to the ideal capacitor.^64^

If *n* = 1, the parameter *n* becomes *Q* = *C*, although *n* typically takes values in the 0 < *n* < 1 range (*n* values can be found in Supporting Information) and is in units of Farads (*F*). The capacitive loop at medium–high frequency is defined in relation to the charge transfer. *R*_2_ represents the charge transfer resistance, and *Q*_2_ or *C*_2_ represents the double-layer capacitance (generated from the dissolution of Al in alkaline solution).^73^ In the low-frequency region, the capacitive loop may be due to surface film formation occurring in the hydrolysis reaction (Al^+^ – Al^3+^ passivation),^74^ represented by *Q*_3_ or *C*_3_, and the associated parallel resistance is represented by *R*_3_. The capacitive and inductive loops are compatible with the frequency-dependent impedance change (Figure 7b) and phase change (Figure 7c). The Bode plot shows that the impedance values of the alloys increase from high to low frequencies, with the 0.0% Ni alloy having a greater impedance than the other Ni-doped alloys. A high shift in the phase angle indicates that the corrosion product layer is dense (like an oxide film), has good insulating properties, and exhibits high charge transfer resistance.^75^ The highest phase shift is observed for the alloy without Ni addition. After corrosion, FESEM images and charge transfer resistance results are quite consistent with this situation. The data from the experiments are simulated with the equivalent circuit using ZMAN Main Software (WonATech Co., Ltd.); the parameters are calculated, and the results are summarized in Table 3. The detailed data table can be accessed in Table S1. The Chi-square value is a parameter that shows the agreement between the experimental data and the simulations. The results found at the levels of 10^–5^ and 10^–6^ are in good agreement with the experimental data and the simulations.

**Table 3:** EIS Simulation Data of Alloys in 3 M NaOH

sample	*R*_s_ (Ω)	*L*_1_ (nH)	*R*_1_ (mΩ)	*Q*_1_ or *C*_1_ (mΩ^–1^ s^n^ or mF)	*R*_2_ (Ω)	*Q*_2_ or *C*_2_ (mΩ^–1^ s^n^ or mF)	*R*_3_ (mΩ)	*Q*_3_ or *C*_3_ (mΩ^–1^ s^n^ or mF)
0.0% Ni	0.88	21.85	178.61	8.51	2.38	0.24	388.54	180.02
0.1% Ni	0.76	44.28	241.79	1.04	1.27	0.30	647.22	73.65
0.5% Ni	1.04	45.73	274.19	2.4	0.52	25.73	29.95	0.17

The charge transfer resistance value is an important parameter in determining the corrosion resistance of the alloys. A low *R*_2_ value suggests that electrochemical corrosion is more likely. The lowest *R*_2_ value is that for the 0.5% Ni alloy. The presence of Ni in the alloy increases corrosion, and these results are in accordance with both polarization and hydrogen generation measurements. The decrease in charge transfer resistance (from 2.38 to 0.52 Ω) is also thought to be related to microstructural transformation from the Al–Si–Ni phase (3.29 μm at 0.1%Ni) to the Al–Ni phase (13.59 μm at 0.5%Ni). With increasing Ni content, growing secondary phase particles form microgalvanic cells at the Al matrix–electrolyte interface. The growth of these particles can increase the active electrochemical surface area by altering the surface morphology. This is quantitatively confirmed by the increase in the amount of *Q*_2_. Furthermore, these secondary phases can act as active centers with low overvoltage for the HER. This is supported by a decrease in the charge transfer resistance (*R*_2_). Parameters related to hydrogen evolution, *L*_1_ and *R*_1_ values, are the largest in the 0.5% Ni alloy. The high inductance value may indicate an increase in local corrosion centers on the surface. The higher *R*_1_ resistance can be explained by the fact that hydrogen bubbles partially mask the surface due to their dense accumulation in these active regions, creating extra insulation and restricting effective ionic transfer. This, in turn, leads to an increase in the electrical resistance on the surface. The presence of a surface oxide film can be monitored through *Q*_3_ or *C*_3_ capacitance. The significant decrease in surface film capacitance (*Q*_3_/*C*_3_ from 180.02 to 0.17 mF) suggests a change in the passive layer’s character with the addition of Ni. It is predicted that the growing secondary phase particles affect the homogeneity of the surface oxide film and weaken the film’s insulating properties. This situation can be interpreted as an effect that supports electrochemical activity, transforming the surface passivation into a more permeable structure with increasing Ni content. Surface images and EDX analysis also indicate that the surface oxide film decreases with Ni addition.

In conclusion, the EIS results show qualitative agreement with the increase in the corrosion current observed in the Tafel analysis. The low error margins in the models indicate that the selected equivalent circuits accurately represent the experimental data and support the proposed mechanism, which posits that Ni-rich secondary phase particles affect both the surface reactivity and hydrogen evolution kinetics.

### 3.6 FESEM Analysis after the Hydrolysis Reaction

Morphological analyses of the alloys after hydrogen generation reactions were carried out to shed additional light on hydrogen generation mechanisms and hydrolysis reactions. Figure 8 shows the surface images after 60 s of immersion in an alkaline medium (3 M NaOH solution). In the FESEM images, only pure water washing and drying were performed to avoid removing the surface oxide layer from the samples, and ultrasonic washing was not applied because of this. A dense oxide layer is observed in the 0% Ni (Figure 8a) alloy, while the density of the oxide layer gradually decreases in the 0.1% Ni (Figure 8b) and 0.5% Ni (Figure 8c) alloys.

**Figure 8:**
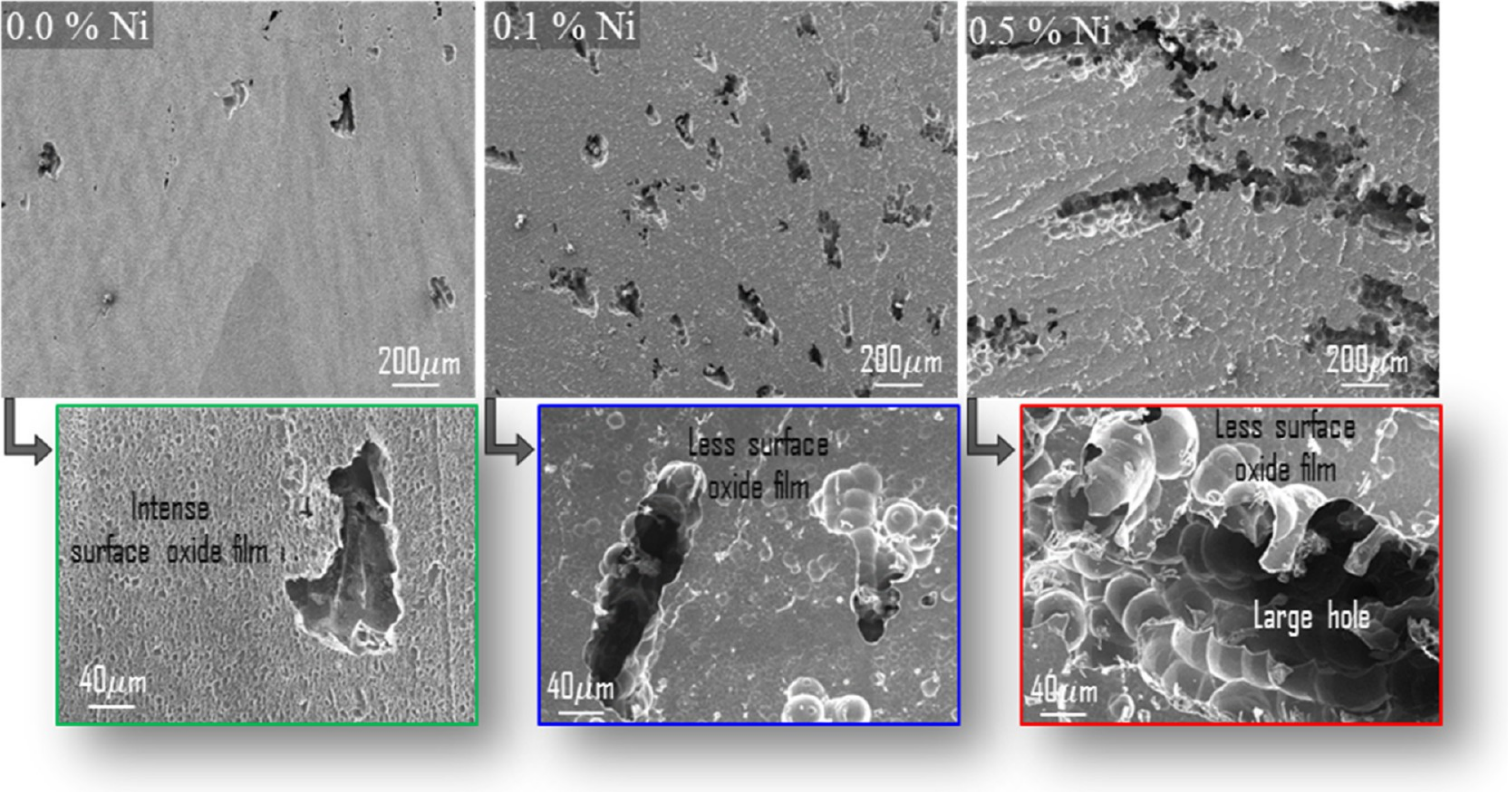
FESEM images of the alloys’ surfaces after corrosion, with enlarged images of the holes.

The effect of alkali corrosion on the surface increases with Ni addition, and even large corrosion holes are formed in the 0.5% Ni alloy. The images clearly show that the dissolution of the alloy increases with the presence of Ni, strongly supporting the hydrogen generation and electrochemical results. In Figure 9, EDX peaks, weight, and atomic percentage composition ratios are given with the green frame representing 0% Ni, the dark-blue frame representing 0.1% Ni, and the red frame representing 0.5% Ni alloy. EDX analysis across selected regions shows that the oxide ratio is highest in the ternary alloy without Ni doping. Moreover, the Ni ratio increases as the Ni doping increases in the quaternary alloys. These results support the comments regarding the density of the surface oxide layer and show that the addition of Ni to the alloy is successful.

**Figure 9:**
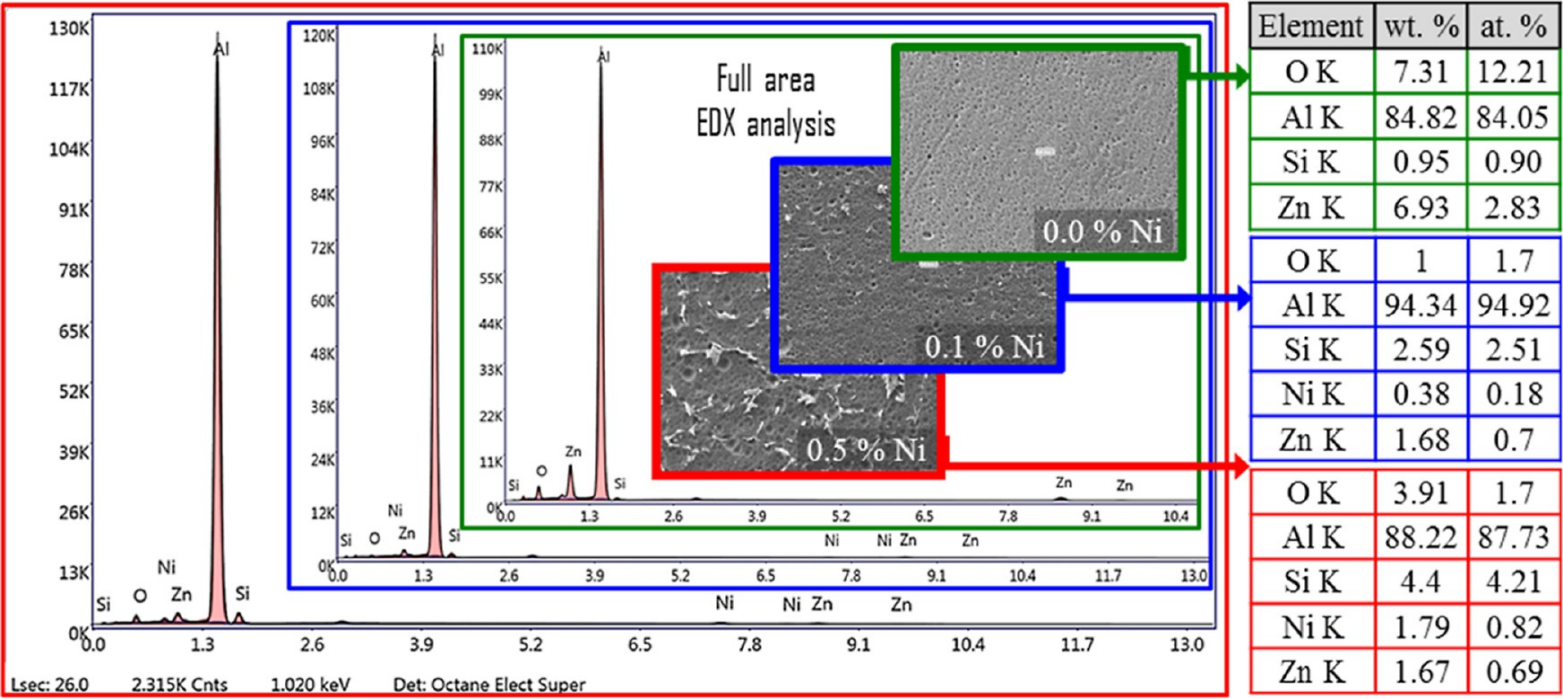
EDX peak graphs and elemental distribution table after alloy surfaces were exposed to a 3 M NaOH solution.

The mapping process was performed on the 0.5% Ni quaternary alloy surface exposed to an alkaline solution. The visual showing the percentage distribution of pixels on the surface according to colors and the mapping colored according to the elements (Al green, Ni fuchsia, Si light orange, O turquoise, and Zn red) is given in Figure 10. Surface O mapping showed that the presence of Ni reduced the surface oxide formation (pay attention to atomic ratios). FESEM-mapping results show that the surface consists of an 87% AlK phase, with 7% unassigned regions due to dissolution. The Ni map indicates that the elongated secondary phase particles remain largely undissolved in the alkaline environment, while the Al map reveals that the matrix surrounding these particles preferentially dissolves. The fact that Ni-rich second-phase particles remain undissolved in the corrosive environment points to the high electrochemical stability of Ni–Al-based phases, consistent with literature findings that phase composition is a key factor in determining corrosion resistance in Ni–Al systems.^76^ The overlap of the oxygen distribution with Al-dissolving regions indicates the accumulation of oxide/hydroxide products in anodic dissolution zones. The Si map, however, separates from Ni-containing regions and concentrates at different locations, supporting the conclusion that the preferential exclusion of Si observed in the precorrosion FESEM-EDX analysis is maintained after corrosion.

**Figure 10:**
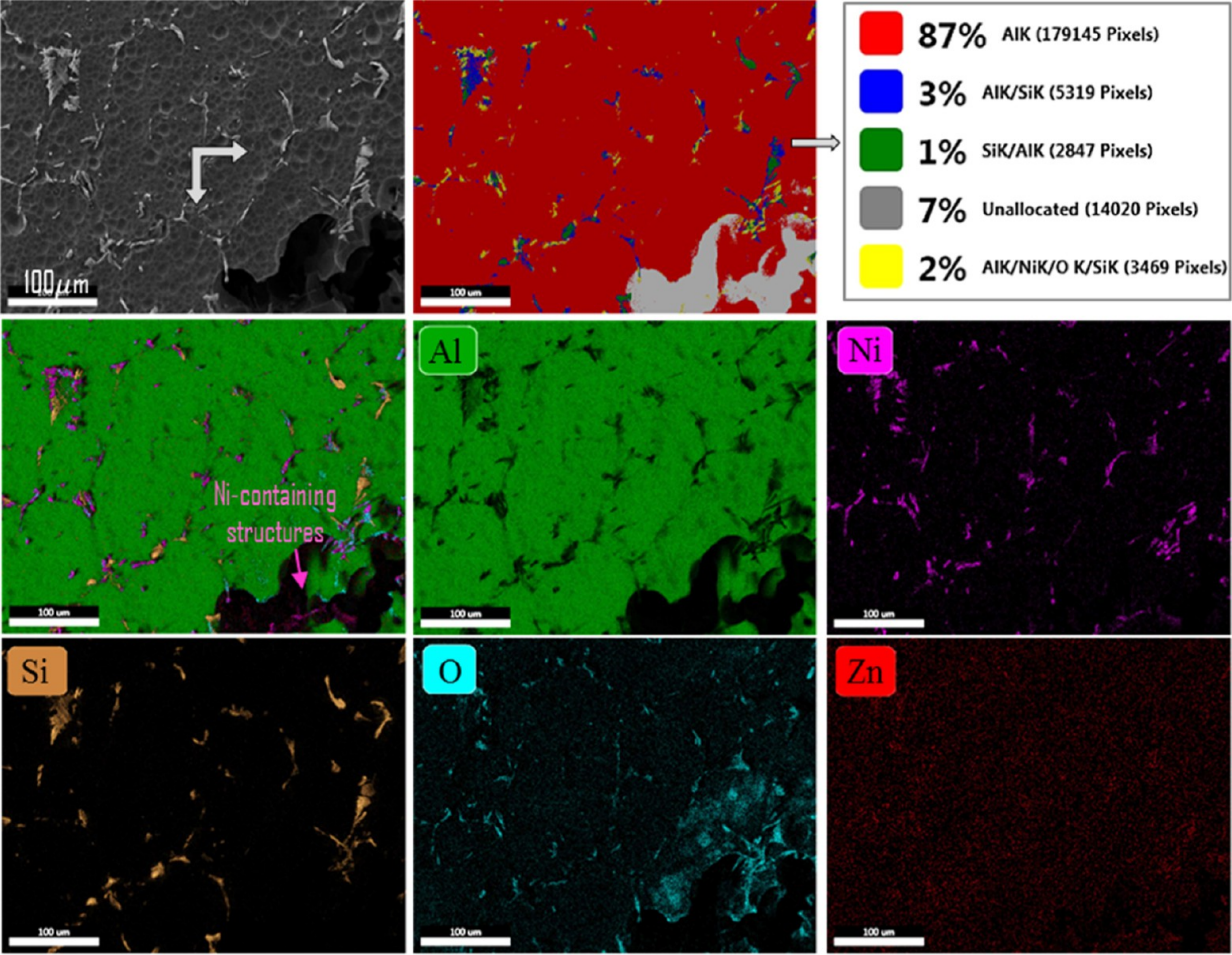
FESEM-mapping analysis of the 0.5% Ni quaternary alloy surface after exposure to alkaline solution.

Furthermore, it is thought that the distribution of elongated second-phase particles within the Al matrix may disrupt the continuity of the surface oxide film.^77^ Thus, it is considered that continuous contact between the fresh solution and the active surface is ensured, and Al dissolution is accelerated, thereby contributing to hydrogen generation.^78^ This suggests that Ni-containing particles in the 0.5% Ni alloy, observed to be ∼ 13.1 μm long in quantitative analysis, function as a cathodic region, while the Al matrix undergoes anodic dissolution to form oxide/hydroxide products. These findings support the idea that changes in phase size and morphology can affect galvanic activation and that the microstructural architecture driving hydrolysis kinetics is largely preserved during corrosion.

The addition of the Ni element improves the hydrogen generation and electrochemical performance with Al–Ni phases. In this newly developed alloy, Ni offers significant support for hydrolysis by highlighting the inherent positive properties of the elements in the alloys. In addition, the contribution of the microgalvanic corrosion mechanism can be observed in samples with corrosion holes in different areas of the sample surface. The possible mechanism is the formation of a potential difference between the phases, originating from the multielement structure of the alloy, which causes galvanic pairs to form. Regarding this, Dolgikh et al.^79^ found that significant potential differences occur between pure Si and pure Al phases, and the Mg adduct can cause microgalvanic corrosion. The formation of microgalvanic cells may cause a regional temperature increase and accelerate the reaction. Another issue is that the alloy’s active ions, such as Zn^2+^, may contribute to reactions at the Al surface during the dissolution–deposition process. In conclusion, these are possible mechanisms explaining the effects of the increasing Ni addition process on hydrogen generation in the Al–Zn–Si–Ni quaternary system. In addition, a schematic representation of the reaction behavior, including a visual illustration of the shape and dimensions of the microstructures and the changes in hydrogen generation under the influence of possible mechanisms, is given in Figure 11. In the diagram, the Al matrix acts as the anode (Al → Al^3+^ + 3e^–^), while the Ni-rich secondary phases act as the catalytic cathode for the hydrogen evolution reaction (2H_2_O + 2e^–^ → H_2_ + 2OH^–^). There is a net flow of electrons from the Al matrix to the secondary phase particles. It represents the microgalvanic interaction supported by EIS and Tafel conclusions.

**Figure 11:**
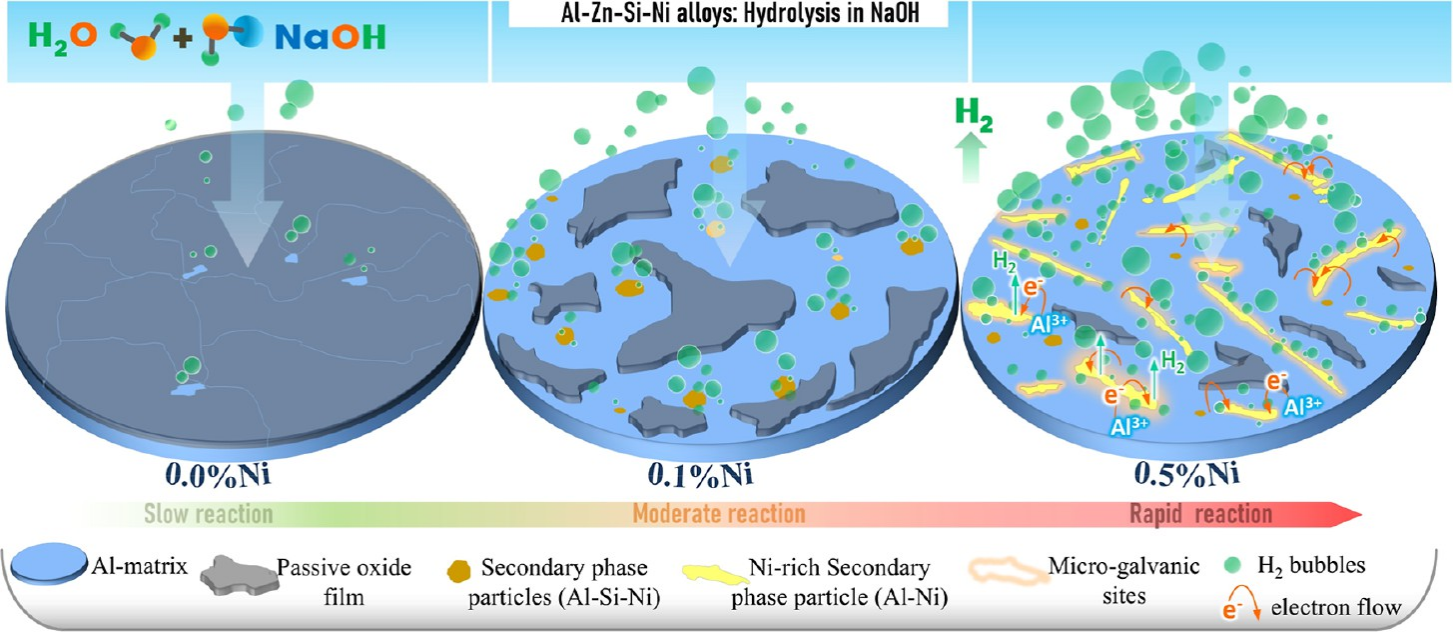
Schematic representation of hydrogen generation from Al–Zn–Si alloys with different Ni additions by hydrolysis in alkaline media (in the diagram, the Al matrix acts as the anode, while the Ni-rich secondary phases act as the catalytic cathode for the hydrogen evolution reaction. There is a representative electron flow from the Al matrix to the Ni-rich secondary phase particles.

In order to compare the results of the hydrogen generation and electrochemical analysis of this study with other scientific studies on Ni-containing Al material for hydrolysis (there are very few studies available), an evaluation table is included in Table 4.

**Table 4:** Comparison with the Literature

material	method	medium	temp.	H_2_ rate	*E*_cor_ (explanation)	*E*_A_ (explanation)	ref.
Al/Ni/NaCl (NaCl = 19 wt %)	ball-milling	NaOH (0.05 M)	30 °C	2.87 mL g^–1^ min^–1^	–1.55 V (vs SCE)	54 kJ mol^–1^ (45–60 °C)	^41^
Al-1wt %Ni	melting	NaOH (5 wt %)	30 °C	8.76 mL cm^–2^ min^–1^	–0.1.19 V (vs SCE)	-	^53^
Al(0.4 g) + Ni–B (0.04 g) catalyst	Al powder + (catalyst)	water	55 °C	0.99 mL g^–1^ min^–1^	-	-	^61^
Al/Ni	ball-milling	NaOH (0.25 M)	70 °C	130 mL g^–1^ s^–1^	–0.57 V (vs Hg/Hg_2_Cl)	-	^62^
Al/Ni	ball-milling	alkali-water (pH:9.6)	35 °C	5 mL g^–1^ min^–1^	–0.46 V (vs SCE)	-	^60^
Al–2Zn–1Si^+^0.5Ni (wt %)	melting alloy	NaOH (3 M)	340 K	85.16 mL g^–1^ min^–1^ 11.86 mL cm^–2^ min^–1^	–1.377 V (vs Ag/AgCl)	17.28 kJ mol^–1^ (330–340 K)	this work

### 3.7 XPS Analysis

Figure 12 shows the XPS survey spectra of the Al–2%Zn–1%Si ternary and 0.5% Ni-doped Al–2%Zn–1%Si quaternary alloy acquired on the surface of NaOH-treated alloys. As seen from Figure 12, Al, Zn, and O elements were observed, but the peaks of the doped Si and Ni atoms were not. Zn- and Al-rich surfaces are formed, and a significant amount of oxide exists. C 1s peaks are also observed, which possibly belong to carbon contamination.

**Figure 12:**
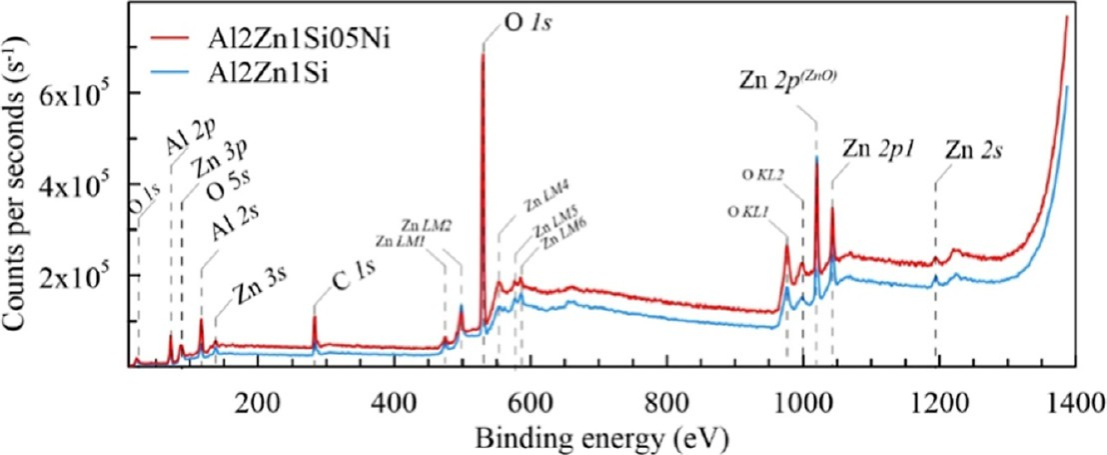
XPS survey spectra of Al–2%Zn–1%Si ternary and 0.5% Ni-doped Al–2%Zn–1%Si quaternary alloys. LM and KL indicate the Auger lines of Zn and O, respectively.

Figure 13a,b shows the experimental A1 2p core-level spectra of the Al–2%Zn–1%Si ternary and 0.5% Ni-doped Al–2%Zn–1%Si quaternary alloys. These spectra showed a close double peak. Each spectrum was deconvoluted into two peaks, and the binding energies were 71.39 and 74.05 eV for the undoped ternary alloy and 71.19 and 74.11 eV for the Ni-doped quaternary alloy. First peaks (fit Al1) at low binding energies (∼72 eV) can be attributed to the presence of Al in metallic form (Al).^30,80,81^

**Figure 13:**
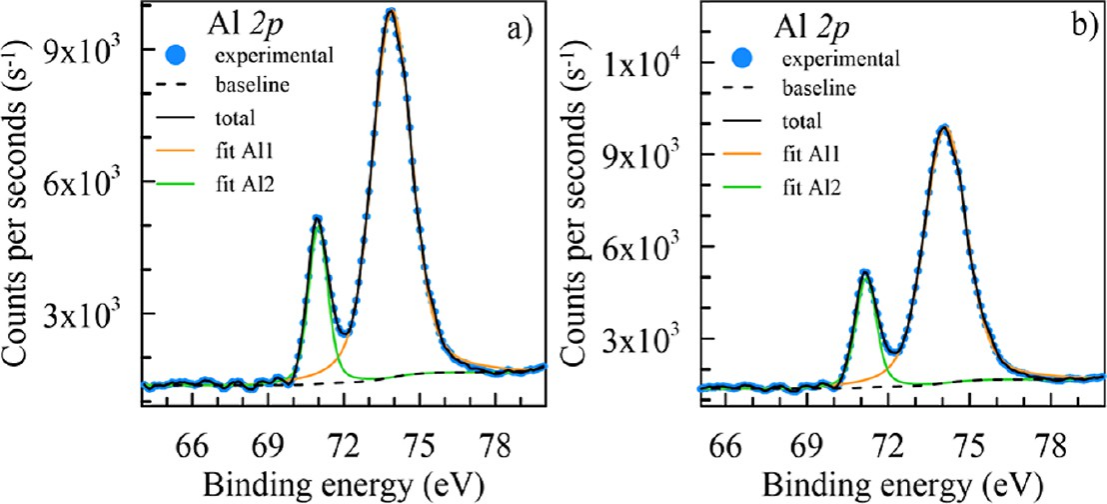
High-resolution Al 2p core-level spectra acquired on the surface of (a) Al–2%Zn–1%Si and (b) 0.5% Ni-doped Al–2%Zn–1%Si alloys.

Second peaks (fit Al2) at higher binding energies (∼74 eV) usually originate from oxidized states of aluminum (Al^3+^), and the form of these states is usually an Al_2_O_3_ or AlOOH layer.^30,82^ The presence of Al^0^ content on the surface of the NaOH-treated alloys indicates that the aluminum oxide film has a greater thickness than that observed in the aluminum layers in Ni-doped alloys. The relative peak area ratio of Al^0^ to Al^3+^ was found to be 21% for the Ni-doped quaternary alloy and 15% for the ternary alloy. This lack of oxidized states also explains a higher H_2_ generation rate in Ni-doped alloys. Figure 14a,b shows the Zn 2p core-level spectra of the Al–2%Zn–1%Si ternary and 0.5% Ni-doped Al–2%Zn–1%Si quaternary alloy. Both spectra have two peaks for the 2p_1/2_ and 2p_3/2_ orbitals in zinc. Each spectrum could be fitted to a single component, and the binding energy values of the 2p_3/2_ orbitals in both spectra were 1022.57 and 1022.89 eV for the Al–2%Zn–1%Si ternary alloy and the 0.5% Ni-doped Al–2%Zn–1%Si quaternary alloy, respectively. Similarly, binding energy values of Zn 2p_1/2_ were determined as 1045.52 and 1045.65 eV for the Al–2%Zn–1%Si ternary alloy and 0.5% Ni-doped Al–2%Zn–1%Si quaternary alloy. This binding energy value corresponds to ZnO^80,81,83^ and ZnAl_2_O_4_.^80,84,85^ Also, the binding energy difference with Zn 2p_1/2_ and 2p_3/2_ is close to 23.2 eV for both alloys and consistent with the literature.^30,82^

**Figure 14:**
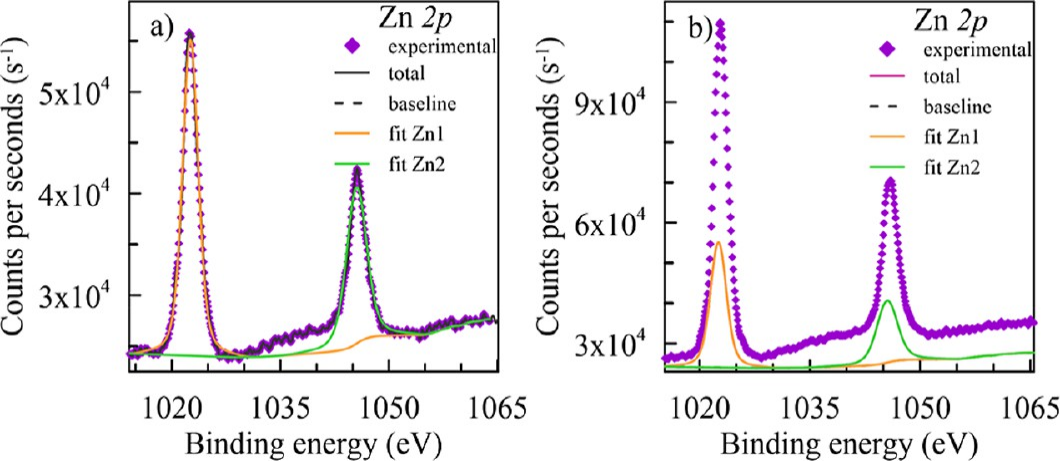
High-resolution Zn 2p core-level spectra acquired on the surface of (a) Al–2%Zn–1%Si and (b) 0.5% Ni-doped Al–2%Zn–1%Si alloys.

Figure 15a,b shows the 1s core-level spectra of Al–2%Zn–1%Si ternary and 0.5% Ni-doped Al–2%Zn–1%Si quaternary alloys. Both spectra have one wide asymmetric peak for the 1s orbital of the oxygen, and deconvolution of the 1s peaks of alloys gives three peaks and confirms the existence of oxides. The most intense peak (at a binding energy of 531.74 and 531.81 eV for ternary and quaternary alloys, respectively) may be associated with the presence of oxygen in the aluminum oxide (Al_2_O_3_).^81,83,86^ Furthermore, less intense components of deconvoluted peaks (fit O1) appear at 530.61 eV for the Al–2%Zn–1%Si ternary alloy, which may be attributed to the presence of O^2–^ ions in the form of zinc oxide and/or silicon oxide. However, this binding energy value shifted toward higher energies (530.80 eV) at the Ni-doped alloy, possibly an effect of oxidized Ni atoms.^83,86,87^ Also, the relatively high energy of these deconvoluted peaks (fit O3) usually indicates the presence of chemically adsorbed or nonlattice oxygen molecules or water molecules.^81,83,86^ Besides all this binding energy information, the atomic percentage ratio of fit O2 and fit O3 (see the inset in Figure 15) decreased, while it increased for fit O1. So, oxidation of metals is directed toward other doping atoms rather than aluminum. Here, the effect of adding Ni atoms to the ternary alloy is clearly observed, and similar behaviors exist in the literature.^86,88^

**Figure 15:**
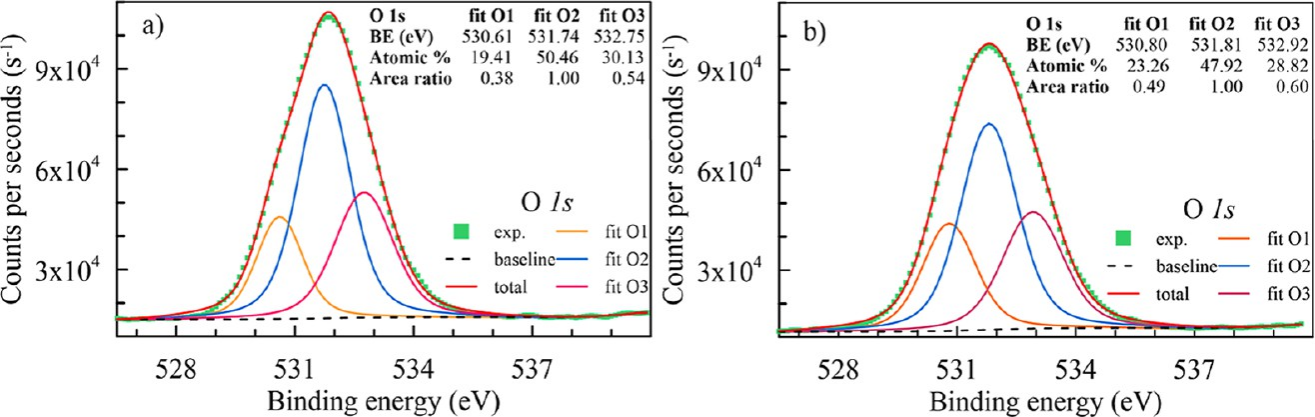
High-resolution O 1s core-level spectra acquired on the surface of (a) Al–2%Zn–1%Si and (b) 0.5% Ni-doped Al–2%Zn–1%Si alloys.

Similarly, Ni 2p and Si 2p core-level spectra (see Figure S2) of Al–2%Zn–1%Si ternary and 0.5% Ni-doped Al–2%Zn–1%Si quaternary alloys have a few peaks, which indicate their own oxidation states. Deconvoluted Ni 2p core-level spectra exhibit characteristic components corresponding to both metallic and oxidized nickel species. The peak centered at 851.46 eV is assigned to metallic Ni^0^ (Ni 2p_3/2_), while the intense satellite feature at 860.64 eV confirms the presence of Ni^2+^ species, characteristic of nickel oxide or nickel hydroxide phases.^89,90^ Additionally, FESEM-EDX mapping images reveal this more clearly.

## 4 Results and Discussion

New quaternary Al–Zn–Si–Ni alloys containing 0.1 and 0.5% Ni at two different weight ratios were developed to investigate the hydrogen generation and electrochemical performance by using the alkaline hydrolysis method.

Quantitative FESEM image analysis showed that with increasing Ni content, the second-phase surface phase ratio increased from 0.38 to 1.65%, and the average particle area increased from 5.24 to 13.14 μm^2^. This revealed that the preferential exclusion of Si led to the formation of a Ni-rich binary phase, thereby enhancing galvanic activation and increasing the hydrogen generation rate. Two different hydrogen generation rate calculations were performed to compare hydrogen generation performance from alloys, namely, *V*_g_ and *V*_s_. At the 340 K reactor set temperature, the *V*_g_ value is 36.89 mL g^–1^ min^–1^ for 0% Ni content and 85.16 mL g^–1^ min^–1^ for 0.5% Ni content, and the *V*_s_ values are 4.66 and 11.86 mL cm^–2^ min^–1^, respectively. All results show that the alloy with 0.5% Ni content has the highest generation rate. In the reaction kinetic analysis, the $\overset{®}{E_{A}\;}$ value, which was 81.91 kJ mol^–1^ for the 0.0% Ni alloy, decreased by approximately one-fifth to 17.28 kJ mol^–1^ for the 0.5% Ni alloy. This indicates that Ni doping is less dependent on the temperature. Ni doping significantly increases the level of activation of Al in alkaline media.

While the Ni-free Al–Zn–Si alloy exhibited passivation, this behavior was not observed in the Ni-added alloys. As the Ni content increased, the corrosion potential shifted to positive values, but the corrosion current density increased, and anodic dissolution accelerated. Polarization, hydrogen production, and EIS results were consistent with the lowest charge transfer resistance being determined in the alloy containing 0.5% Ni. Ni reduced the capacitance value by thinning the surface oxide layer and increasing the corrosion. XPS analyses showed that Ni reduced the proportion of oxidized Al (Al^3+^) and increased the amount of metallic Al (Al^0^). Deconvoluted Ni 2p core-level spectra exhibit characteristic components corresponding to both metallic and oxidized nickel species. Consequently, Ni addition enhances the hydrogen generation performance by increasing the electrochemical activity of the alloys. Postcorrosion FESEM and EDX analyses revealed the effects of the corrosion process. Al–Ni and Al–Ni–Si phases can accelerate Al dissolution, leading to additional hydrogen generation. Specifically, it was found that the Ni-containing secondary phase particles in the 0.5% Ni alloy act as a cathodic region, while the Al matrix undergoes anodic dissolution to form oxide/hydroxide products. It was concluded that changes in phase size and morphology can affect galvanic activation and that the microstructural architecture driving hydrolysis kinetics is largely preserved during corrosion. As a result, the observed phenomena may explain the effects of the alloying process on Al-water-based hydrogen generation via hydrolysis. Fundamental microstructural and electrochemical findings from such casting-produced alloys have the potential to provide insights into alternative production methods such as mechanical alloying (ball milling) and rapid solidification.

## Supplementary Material



## Data Availability

The data underlying this study are available in the published article and its online Supporting Information.

## Notes

Reactor temperature, EIS simulation data, additional XPS graphs, and quantitative morphological analysis data (PDF)
