# Supplementary material for: Ni-Induced Novel Multi-Al-Alloys for Efficient and On-Demand Hydrolysis-Based Hydrogen Generation
Source: ACS Omega. 2026 May 5;11(19):28817–33. doi: 10.1021/acsomega.6c01514 (PMC13191673; doi:10.1021/acsomega.6c01514)
Supplement: Supplementary file 1 [file ao6c01514_si_001.pdf]

Supporting Information

Ni-induced novel multi-Al-alloy for efficient and on-demand hydrolysis-based hydrogen generation

Osman Kahveci [1,2,3*], Tuncay Karaaslan [1] and Abdullah Akkaya [2,4]

[1]Erciyes University, Faculty of Science, Physics Department, 38039, Kayseri, Türkiye

[2]Erciyes University METALION Research Group, 38039 Kayseri, Türkiye

[3]Erciyes University Energy Conversion Research and Application Center, 38039 Kayseri, Türkiye

[4]Mucur Technical Vocational Schools, Tech. Prog. Department, Kırşehir Ahi Evran University, 40500 Kırşehir, Türkiye

* Corresponding author. Tel.: +903522076666-33138; Fax: +903524374933

Email: kahveci@erciyes.edu.tr

Reactor temperature while the reaction is in progress

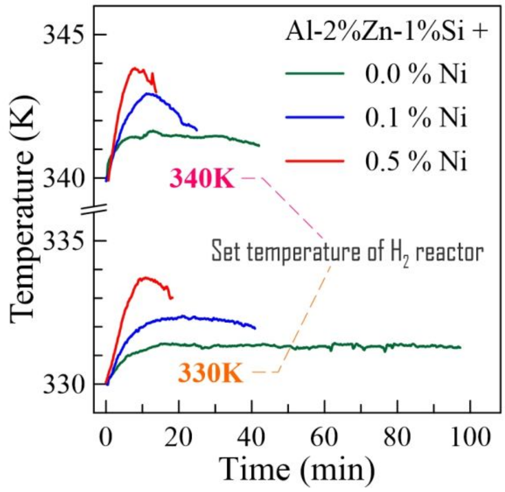

Figure S1. Variation of reactor temperatures of alloys over time for two different set temperatures (330K and 340K).

As shown in Figure S1, the reactor temperature variation over time during hydrogen generation. It is possible to see the effect of the exothermic reaction (Al with NaOH solution) from these graphs. The reactor temperature, which starts at 330 K, reaches approximately 331.4 K in 15 minutes in the 0.0% Ni-doped alloy, while it reaches 333.8 K in 10.8 minutes in the 0.5% Ni-doped alloy. Similarly, the reactor temperature, starting at 340 K, reaches 341.6 K in the 0.0% Ni alloy and 343.8 K in the 0.5% Ni alloy in a shorter time.

EIS simulation data

The data obtained from the experimental EIS results were simulated with the equivalent circuit using the ZMAN™ Master Software (WonATech Co., Ltd.) and the parameters were calculated, and the results are included in the detailed data table in Table S1.

Table S1. EIS simulation data of alloys in 3 M NaOH.

Sample	R_s (Ω)	L_1 (nH)	R_1 (mΩ)	Q_1 or C_1 (m$\Omega^{-1}s^n$ or mF)	n_1	R_2 (Ω)	Q_2 or C_2 (m$\Omega^{-1}s^n$ or mF)	n_2	R_3 (mΩ)	Q_3 or C_3 (m$\Omega^{-1}s^n$ or mF)	n_3	Chi-sqr
**0.0 % Ni**	0.88	21.85	178.61	8.51	0.72	2.38	0.24	1	388.54	180.02	1	2.9×10^{-5}
**0.1 % Ni**	0.76	44.28	241.79	1.04	0.74	1.27	0.30	0.94	647.22	73.65	0.86	6.8×10^{-6}
**0.5 % Ni**	1.04	45.73	274.19	2.4	1	0.52	25.73	0.64	29.95	0.17	1	2.8×10^{-6}

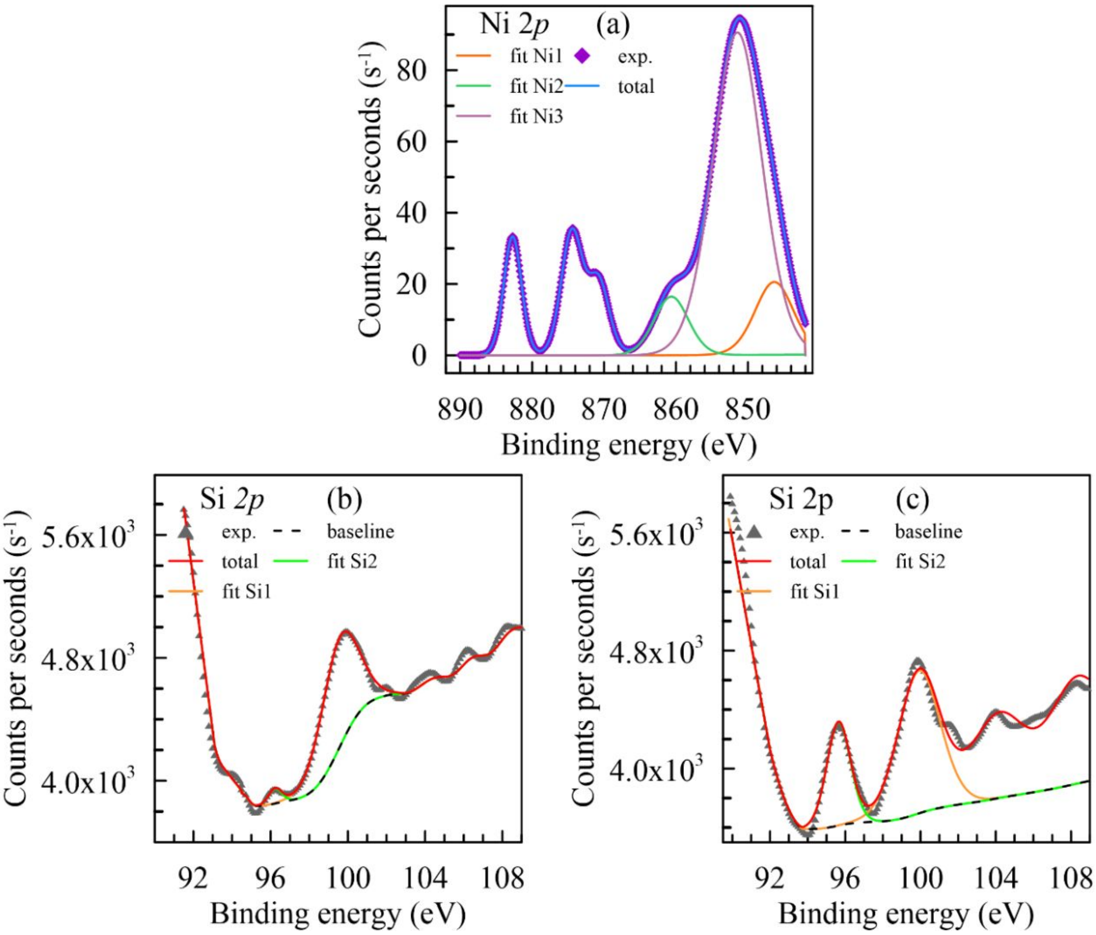

Figure S2. High-resolution (a) Ni *2p* and (b-c) Si *2p* core level spectra acquired on the surface of Al-2% Zn-1% Si and 0.5% Ni doped Al-2% Zn-1% Si alloy, respectively.

Ni *2p* core level spectra of 0.5% Ni-doped Al-2% Zn-1% Si quaternary alloy (Figure S2a). peak intensity was very low. Deconvoluted Ni *2p* core level spectra exhibits characteristic components corresponding to both metallic and oxidized nickel species. The peak centered at 851.46 eV is assigned to metallic Ni^0 (Ni $2p_{3/2}$), while the intense satellite feature at 860.64 eV confirms the presence of Ni^{2+} species, characteristic of nickel oxide or nickel hydroxide phases [1-2]. In the higher binding energy region, the peaks located at 870.8 and 874.5 eV are attributed to the Ni $2p_{1/2}$ components of metallic and oxidized nickel, respectively, whereas the feature at 882.7 eV corresponds to the shake-up satellite of Ni^{2+} [2-3]. The low binding energy component observed at 846 eV is likely associated with Ni LMM Auger overlap or an asymmetric metallic tail contribution [4]. Similarly, Figure S2 b-c shows the Si *2p* core level spectra of Al-2% Zn-1% Si ternary and 0.5% Ni doped Al-2% Zn-1% Si quaternary alloys, respectively. These peaks

possibly contain other phase signals formed by metals in these alloys, but excessive oxide states masked them, as mentioned in FESEM results.

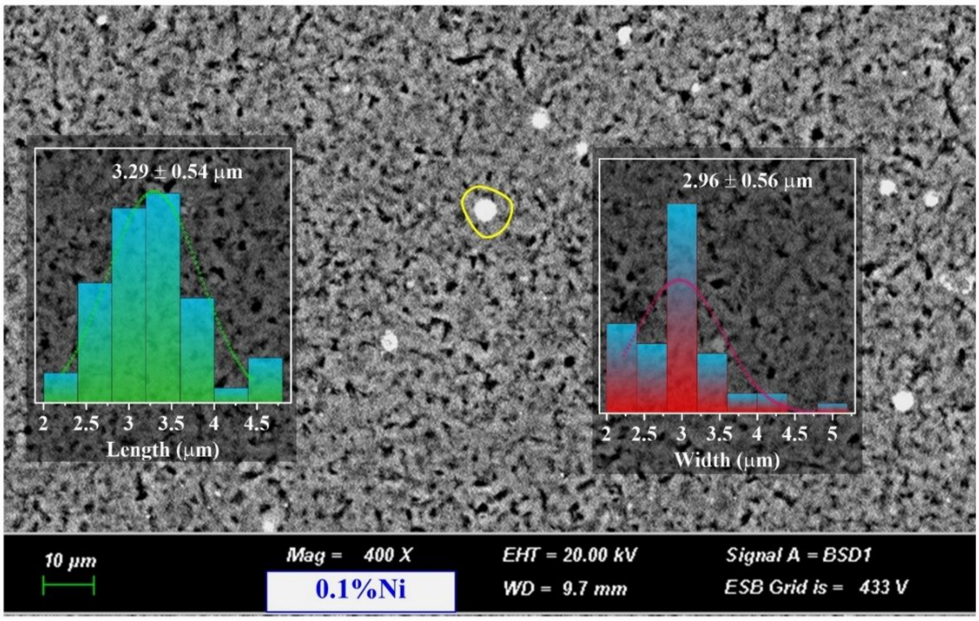

Figure S3. Dimensions of microstructures measured from FESEM surface image of 0.1% Ni alloy.

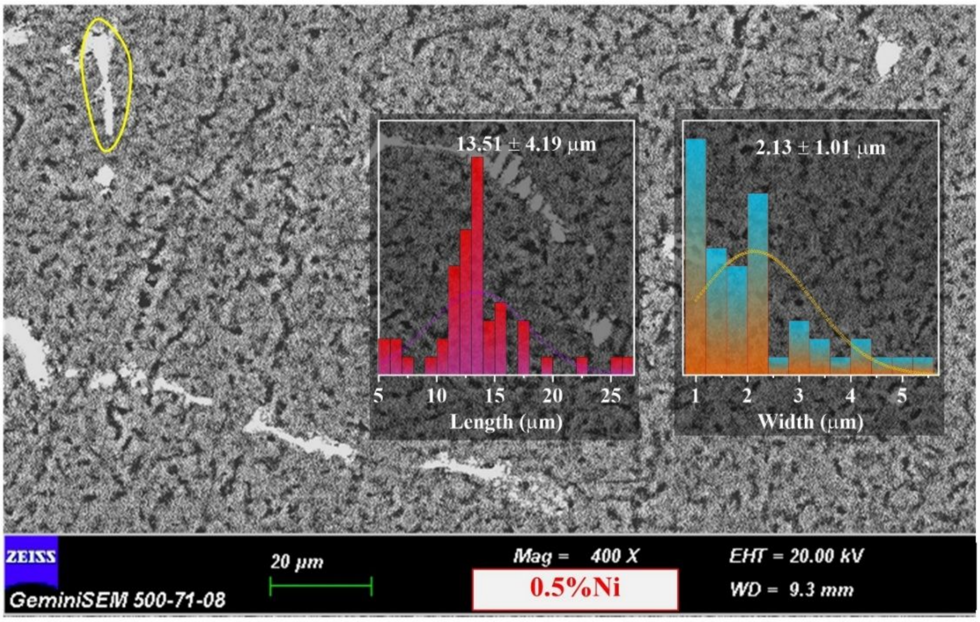

Figure S4. Dimensions of microstructures measured from FESEM surface image of 0.5% Ni alloy.

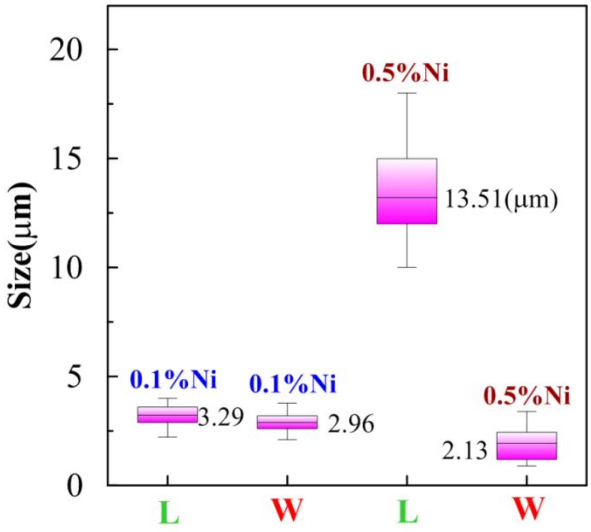

Figure S5. Comparative representation of width-length measurements of microstructures of 0.1%Ni and 0.5%Ni alloys.

Although the increment in Ni addition did not cause a significant change in the width of the microstructures, it increased their length by approximately four times. Ni addition significantly contributed to hydrogen production by adjusting the size and shape of the microstructures.

Table **S2.** Quantitative characterization of secondary phases for 0.1%Ni and 0.5%Ni alloys

Parameter	0.1%Ni Alloy	0.5%Ni Alloy
Particle Count	44	76
Total area (μm^2)	230.7	998.2
Average particle size (μm^2)	5.2	13.1
Area fraction (%)	0.38	1.65

References

1. Grosvenor, A. P.; Biesinger, M. C.; Smart, R. S. C.; McIntyre, N. S., New interpretations of XPS spectra of nickel metal and oxides. *Surf Sci* **2006,** *600* (9), 1771-1779.
2. Guzman-Bucio, D. M.; Gomez-Sosa, G.; Cabrera-German, D.; Torres-Ochoa, J. A.; Bravo-Sanchez, M.; Cortazar-Martinez, O.; Carmona-Carmona, A. J.; Herrera-Gomez, A., Detailed peak fitting analysis of the Ni 2p photoemission spectrum for metallic nickel and an initial oxidation. *J Electron Spectrosc* **2023,** *262*, 147284.
3. Fu, Z.; Hu, J.; Hu, W.; Yang, S.; Luo, Y., Quantitative analysis of Ni^{2+}/Ni^{3+} in $Li[Ni_xMn_yCo_z]O_2$ cathode materials: Non-linear least-squares fitting of XPS spectra. *Appl Surf Sci* **2018,** *441*, 1048-1056.
4. Bennett, P. A.; Fuggle, J. C.; Hillebrecht, F. U.; Lenselink, A.; Sawatzky, G. A., Electronic structure of Ni and Pd alloys. III. Correlation effects in the Auger spectra of Ni alloys. *Phys Rev B* **1983,** *27* (4), 2194-2209.
